# Oral Anticoagulants in Patients With Atrial Fibrillation and Advanced or End-Stage Renal Disease: A Systematic Review and Meta-Analysis

**DOI:** 10.31083/RCM52237

**Published:** 2026-09-16

**Authors:** Stylianos Fiflis, Georgios Aletras, Maria Bachlitzanaki, Emmanouil Foukarakis

**Affiliations:** ^1^Department of Cardiology, Venizelio General Hospital of Heraklion, 71409 Heraklion, Greece; ^2^Second Department of Internal Medicine, Venizelio General Hospital of Heraklion, 71409 Heraklion, Greece

**Keywords:** atrial fibrillation, renal insufficiency, chronic, kidney failure, chronic, renal dialysis, peritoneal dialysis, anticoagulants, factor Xa inhibitors, warfarin, stroke

## Abstract

**Background:**

Patients with atrial fibrillation (AF) and advanced chronic kidney disease (CKD) face a markedly elevated risk of both thromboembolic and bleeding events. While oral anticoagulation is the cornerstone of stroke prevention in AF, the optimal use of this treatment in patients with stage 4–5 CKD and end-stage renal disease (ESRD) undergoing dialysis remains uncertain, as these populations were largely excluded from the landmark trials that established the efficacy of direct oral anticoagulants (DOACs) in the general AF population. This study aimed to systematically evaluate and compare the efficacy and safety of vitamin K antagonists (VKAs) and DOACs, including factor Xa inhibitors, compared with alternative anticoagulation strategies or no anticoagulation in patients with AF and advanced CKD, including both non-dialysis-dependent stage 4–5 CKD and dialysis-dependent ESRD.

**Methods:**

A systematic search of PubMed (MEDLINE), the Cochrane Library, and ClinicalTrials.gov was performed through January 30, 2026. Randomized controlled trials and observational cohort studies evaluating oral anticoagulation in adults with AF and stage 4–5 CKD (estimated glomerular filtration rate (eGFR) <30 mL/min/1.73 m^2^, not on dialysis) or ESRD on dialysis were included. Outcomes of interest were a composite of stroke, transient ischemic attack or systemic embolism, major bleeding (International Society on Thrombosis and Haemostasis (ISTH) or study-defined), and all-cause mortality. Owing to clinical and methodological heterogeneity, evidence was synthesized narratively for most comparisons; randomized trials comparing factor Xa inhibitors with VKAs in dialysis-dependent patients were pooled in a random-effects meta-analysis.

**Results:**

A total of 20 studies met the inclusion criteria, comprising four randomized trials, one subgroup analysis of an RCT (ARISTOTLE), and fifteen observational cohorts. Four studies evaluated patients with advanced CKD not receiving dialysis, while sixteen focused on dialysis populations. In non-dialysis stage 4–5 CKD, DOAC therapy, particularly apixaban, was generally associated with comparable or reduced thromboembolic risk and lower rates of major bleeding compared with warfarin. Observational data also suggested a potential mortality benefit with DOACs compared with no anticoagulation. In contrast, findings in dialysis-dependent ESRD were heterogeneous. Warfarin was not consistently associated with reduced stroke risk compared with no anticoagulation and was frequently linked to elevated bleeding risk. Observational studies suggest that apixaban may confer a lower risk of major bleeding compared with VKAs while maintaining similar thromboembolic protection; however, randomized evidence has been limited and underpowered to demonstrate a definitive net clinical benefit. In a meta-analysis of the four dialysis randomized trials (n = 486), factor Xa inhibitors were associated with less ISTH major bleeding than VKAs (pooled risk ratio (RR) 0.64, 95% confidence interval (CI) 0.42–0.99; not retained in Mantel–Haenszel or Hartung–Knapp sensitivity analyses), with no significant difference in stroke, transient ischemic attack or systemic embolism (pooled RR 0.46, 95% CI 0.20–1.02) or all-cause mortality (RR 0.88, 95% CI 0.58–1.35).

**Conclusion:**

The risk–benefit profile of oral anticoagulation differs across the CKD spectrum. In stage 4–5 CKD not requiring dialysis, DOACs appear to offer a more favorable safety profile than VKAs while preserving efficacy. In dialysis-dependent ESRD, the net clinical benefit of anticoagulation remains uncertain, particularly for warfarin. Further adequately powered randomized trials are needed to clarify optimal anticoagulation strategies in this high-risk population. Until then, treatment decisions should remain individualized, balancing thromboembolic and bleeding risk.

**PROSPERO Registration:**

https://www.crd.york.ac.uk/PROSPERO/view/CRD420261286449.

## 1. Introduction

Atrial fibrillation (AF) is the most common cardiac arrhythmia worldwide and is associated with an increased risk of ischemic stroke and systemic thromboembolism. The pathophysiology of thrombus formation in AF is primarily driven by blood stasis in the left atrial appendage, endothelial dysfunction, and a systemic prothrombotic state, fulfilling the components of Virchow’s triad. In population-based cohorts, the lifetime risk of developing AF approaches 20% in individuals without comorbidities and exceeds one-third among those with predisposing conditions such as hypertension, diabetes mellitus or chronic kidney disease (CKD) [1,2,3].

CKD and AF frequently coexist, sharing a bidirectional relationship where each condition exacerbates the progression of the other. In CKD, persistent activation of the renin–angiotensin–aldosterone system and sympathetic nervous system leads to oxidative stress, inflammation, and fluid retention. These processes increase atrial pressure, promote fibrosis, and alter ion channel function, resulting in both structural and electrical remodeling of the atria that favor AF development. Additionally, poor blood pressure control and left ventricular hypertrophy, which are common in CKD, worsen diastolic dysfunction and contribute further to atrial enlargement and arrhythmogenic remodeling [3,4,5].

Patients with advanced CKD (Stage 4) and end-stage renal disease (ESRD; Stage 5) face a markedly elevated risk of both thrombotic and bleeding events. Their management demands careful balancing between preventing thrombosis and minimizing hemorrhage, as renal dysfunction concurrently enhances predisposition to both. Uremia induces platelet dysfunction and impairs vascular–platelet interactions, thereby increasing bleeding tendency, whereas chronic inflammation, endothelial activation, and reduced clearance of procoagulant factors contribute to a hypercoagulable state [6,7,8,9].

Oral anticoagulation remains the cornerstone of stroke prevention in patients with AF. Current therapeutic options include vitamin K antagonists (VKAs) and direct oral anticoagulants (DOACs). While DOACs have largely supplanted VKAs in the general AF population due to their predictable pharmacokinetics, fewer drug-drug interactions and lower risk of intracranial hemorrhage, their use in patients with advanced CKD and ESRD is supported by weaker guideline recommendations. Conversely, VKAs, though widely used, pose challenges related to narrow therapeutic windows, variable dosing requirements, and increased risk of both bleeding and vascular calcification in this group. Importantly, patients with severe renal impairment were systematically excluded from pivotal DOAC trials, leaving a significant evidence gap regarding optimal anticoagulant selection in this population. Consequently, current professional guidelines offer no firm recommendations, emphasizing individualized, multidisciplinary decision-making based on thromboembolic and bleeding risk assessment [10,11].

In this systematic review and meta-analysis, we systematically searched randomized controlled trials and observational studies evaluating oral anticoagulants versus alternative anticoagulants or no anticoagulation in patients with advanced CKD or ESRD and AF, in order to identify optimal stroke prevention strategies in this challenging high-risk population.

## 2. Methods

### 2.1 Aim of the Review

Current guidelines provide limited recommendations supported by high-quality evidence on anticoagulation strategies for AF in patients with advanced (CKD stages 4–5, estimated glomerular filtration rate [eGFR] <30 mL/min/1.73 m^2^, not on dialysis) or ESRD [12,13]. This systematic review synthesized evidence from randomized controlled trials (RCTs) and observational studies comparing (1) vitamin K antagonists versus direct oral anticoagulants, (2) vitamin K antagonists versus no anticoagulation, or (3) direct oral anticoagulants versus no anticoagulation, to identify optimal antithrombotic strategies for this vulnerable population.

### 2.2 Study Inclusion Criteria and PICO (Population/Intervention/Comparator/Outcomes) Framework

Eligible study designs included RCTs, prospective and retrospective cohort studies that were published in English. Studies were eligible for inclusion if they compared the effectiveness or safety of different oral anticoagulation regimens, as defined in the PICO framework, in patients with AF and advanced CKD or ESRD on dialysis (hemodialysis or peritoneal), reporting one or more prespecified efficacy or safety outcomes. The PICO framework guiding eligibility is summarized below.

Population (P): Adults (≥18 years) with AF and advanced CKD (stage 4–5, eGFR <30 mL/min/1.73 m^2^, not on dialysis) or ESRD on dialysis (hemodialysis or peritoneal dialysis).

Intervention (I): Oral anticoagulation with DOACs (apixaban, rivaroxaban, dabigatran, edoxaban) at standard or reduced doses or VKAs (warfarin, acenocoumarol, phenprocoumon).

Comparator (C): Other anticoagulants (e.g., VKA vs DOAC, different DOAC doses/regimens) or no anticoagulation (placebo, no therapy).

Outcomes (O): Ischemic outcomes, including ischemic stroke, transient ischemic attack (TIA), myocardial infarction (MI), and other systemic arterial emboli, as reported by included studies. Safety outcomes, including major bleeding per International Society on Thrombosis and Haemostasis (ISTH) definition, or study-specific equivalents including fatal or site-specific bleeds (e.g., intracranial hemorrhage, gastrointestinal bleeding), bleeding that required intervention or hospitalization, or other reported categories, as well as all-cause mortality.

Exclusion criteria comprised pediatric patients (<18 years); studies restricted to CKD stages 1–3 or studies without separate analyses for stages 4–5/ESRD; non-human studies; case reports, editorials, commentaries, or reviews; and studies lacking relevant outcome data.

### 2.3 Search Strategy

The literature was systematically searched in PubMed (MEDLINE) with a final search date of January 30, 2026. The primary PubMed search combined terms for atrial fibrillation, advanced kidney disease, and oral anticoagulants, yielding 1186 records after applying clinical trial and observational study filters. Embase was not searched due to restricted institutional access. This was addressed through supplementary manual searches of reference lists of all the included studies and of relevant reviews and meta-analyses, and targeted ClinicalTrials.gov and Cochrane Library queries. These efforts identified three additional records, which met the inclusion criteria. After deduplication and title/abstract screening per predefined criteria, twenty studies were included in the qualitative synthesis. The Preferred Reporting Items for Systematic Reviews and Meta-Analyses (PRISMA) flow diagram is shown in Fig. 1.

**Fig. 1.**
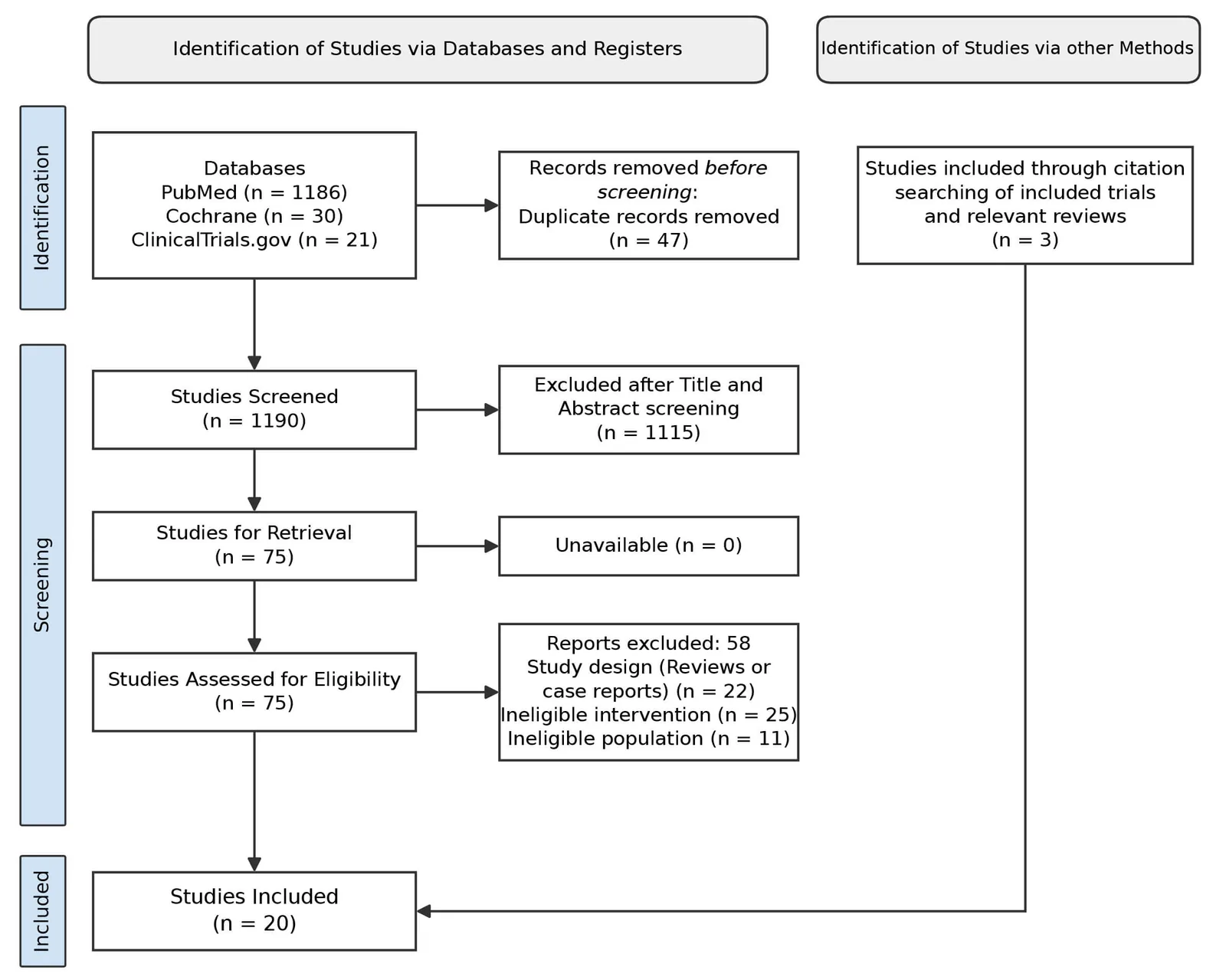
**PRISMA flowchart (Preferred Reporting Items for Systematic Reviews and Meta-Analyses) of study identification, screening, eligibility assessment and inclusion**.

### 2.4 Data Extraction

Two reviewers independently extracted data from the 20 included studies using a standardized form, capturing trial details (design, country, follow-up duration), baseline demographics and patient characteristics (age, sex, dialysis modality, CHA_2_DS_2_-VASc or CHADS2 score [CHA_2_DS_2_-VASc and CHADS2 score are stroke risk assessment score for patients with atrial fibrillation, higher scores correspond to higher stroke risk], comorbidities), intervention specifics (oral anticoagulant [OAC] type/dose, concomitant antiplatelets), and prespecified outcomes with effect measures (hazard ratios [HRs] and 95% confidence intervals [CIs] for stroke/systemic embolism, major bleeding, all-cause mortality; absolute event rates when reported). The tables that were created using the aforementioned data are presented in the results section. Any discrepancies were resolved by consensus or third-reviewer consultation.

### 2.5 Quality Assessment

Risk of bias was independently assessed by two reviewers using validated tools appropriate to study design. RCTs were evaluated with the Cochrane Risk of Bias 2 (RoB 2) tool, examining domains of randomization process, deviations from intended interventions, missing outcome data, outcome measurement, and selection of reported results [14]. Non-randomized observational studies were appraised using the Newcastle-Ottawa scale (NOS) and were assigned stars across three categories, with a maximum score of nine points [15]. Disagreements were resolved by discussion or third-reviewer consultation. Risk of bias assessment is presented in **Supplementary Materials**.

### 2.6 Data Synthesis

Evidence was synthesized narratively due to clinical (dialysis vs non-dialysis CKD, DOAC type/dose variations) and methodological heterogeneity (observational designs, RCTs, variable follow-up) precluding meta-analysis of the overall, clinically heterogeneous evidence base. Studies were grouped by treatment regimens compared (DOACs vs VKAs; OAC vs no OAC) and CKD stage, with effect estimates (HRs with 95% CIs) and event rates for stroke, MI, systemic embolism, ISTH or study-defined major bleeding, and mortality tabulated in tables that are presented in the Results section.

In addition, for the most clinically homogeneous comparison, namely the randomized trials of factor Xa inhibitors versus VKAs in dialysis-dependent patients, a quantitative synthesis was performed [16,17,18,19]. Trial-level event counts for the composite of stroke (ischemic or hemorrhagic), transient ischemic attack and systemic embolism, ISTH major bleeding, and all-cause mortality were extracted for both treatment arms; for the three-arm trial by De Vriese et al. [17], the two rivaroxaban arms were combined and compared with the single VKA arm. Risk ratios (RRs) with 95% CIs were pooled using an inverse-variance random-effects model (DerSimonian–Laird estimator), with a 0.5 continuity correction applied to studies containing a zero-event cell. Statistical heterogeneity was quantified using the I^2^ statistic and Cochran’s Q test. A sensitivity analysis restricted to the trials judged to be at low risk of bias on the Cochrane RoB 2 tool was performed to assess the robustness of the pooled estimates. Pooling was deliberately restricted to this subset, as the remaining comparisons (non-dialysis advanced CKD, and warfarin versus no anticoagulation) comprised too few and too clinically heterogeneous studies to combine. Analyses were performed in Python (version 3.12.3; Python Software Foundation, Wilmington, DE, USA) using the NumPy (version 2.4.4; NumPy Developers; https://numpy.org) and SciPy (version 1.17.1; SciPy Developers; https://scipy.org) libraries.

## 3. Results

### 3.1 Study Characteristics

A total of 20 studies evaluating oral anticoagulation in patients with advanced CKD were included in the qualitative synthesis. Among these, four studies involved patients with advanced CKD not receiving dialysis, while sixteen studies focused on patients undergoing dialysis. The included studies were conducted across multiple regions including North America, Europe, and Asia. Study designs comprised RCTs, prospective cohorts, and retrospective observational cohorts. Most of the included studies were retrospective observational cohort studies, while a smaller number were prospective studies or randomized trials.

Follow-up duration varied considerably between studies, ranging from 26 weeks to several years. Several observational cohorts employed propensity score matching or inverse probability of treatment weighting (IPTW) to reduce baseline differences between treatment groups.

The main study characteristics are summarized in Table 1 (Ref. [16,17,18,19,20,21,22,23,24,25,26,27,28,29,30,31,32,33,34,35]).

**Table 1.** **Study design, setting and follow up duration**.

Trial	Country	Study design	Follow up duration
Advanced CKD patients (Stage 4 and 5)			
Stanifer et al., 2020 [20]	International, 39 countries	RCT subgroup analysis (ARISTOTLE trial)	Median 1.8 years
Ballegaard et al., 2024 [21]	Denmark	Observational retrospective cohort	Median 5.7 years (IQR 3.5–7.6)
Hsu et al., 2023 [22]	Taiwan	Observational retrospective cohort	Max 8 years
Weir et al., 2020 [23]	US	Observational retrospective cohort	Mean 389 days
		Propensity score matched	Max 730 days
Patients on dialysis			
Pokorney et al., 2022 [16]	US	Prospective, open-label, non-inferiority RCT	340 days
De Vriese et al., 2021 [17]	Belgium	Prospective, open-label, superiority RCT	Median 1.88 years
Harel et al., 2025 [18]	Canada and Australia	Prospective, open-label, pilot RCT	26 weeks
Reinecke et al., 2023 [19]	Germany	Prospective, open-label, non-inferiority RCT	37–1370 days (median 429)
Roh et al., 2024 [24]	South Korea	Observational retrospective cohort	Median 3.2 years
Phan et al., 2019 [25]	US	Observational retrospective cohort	2 years
Moore et al., 2024 [26]	US	Observational retrospective cohort	2 years
Siontis et al., 2018 [27]	US	Observational retrospective cohort	5 years (until study end) or death of the patient
Sánchez Soriano et al., 2018 [28]	Spain	Observational retrospective cohort	Max 1159 days
Yodogawa et al., 2016 [29]	Japan	Observational retrospective cohort	Mean 47 months
Mavrakanas et al., 2020 [30]	US	Observational retrospective cohort	Followed up to death, kidney transplantation, or August 2017
Yoon et al., 2017 [31]	South Korea	Observational retrospective cohort	15.9 ± 11.1 months
		Propensity score matched	
Genovesi et al., 2017 [32]	Italy	Observational prospective cohort	4 years
Chan et al., 2015 [33]	US	Observational retrospective cohort	2 years
Wakasugi et al., 2014 [34]	Japan	Observational prospective cohort	Followed for a total of 110 person years
Shah et al., 2014 [35]	Canada	Observational retrospective cohort	NR

CKD, Chronic Kidney Disease; US, United States; IQR, interquartile range; RCT, Randomized Controlled Trial; NR; Not Reported.

### 3.2 Risk of Bias Assessment

Methodological quality and risk of bias were assessed for all included studies. Among the cohort studies, the majority demonstrated a low risk of bias according to the NOS, with only two studies reaching a moderate-quality rating (NOS score of 5 or 6). Regarding the four randomized controlled trials and the ARISTOTLE subgroup analysis, all evaluated via the Cochrane RoB 2 tool, two were classified as having a low risk of bias, while the remaining three were judged to have ‘some concerns’ due to potential bias within a single domain. Detailed, domain-level assessment tables for all included studies are provided in the **Supplementary Material**.

### 3.3 Patient Demographics and Renal Function Characteristics

Four studies evaluated patients with advanced CKD not receiving dialysis, including the studies by Stanifer et al. [20], Ballegaard et al. [21], Hsu et al. [22], and Weir et al. [23]. In these studies, patient age was generally advanced, with median or mean ages ranging from approximately 78 to 84 years. The proportion of male participants ranged from approximately 38% to 58% across treatment groups [20,21,22,23].

Different anticoagulation strategies were compared across studies. The study by Stanifer et al. [20] compared apixaban (5 mg or 2.5 mg) with warfarin, while Ballegaard et al. [21] evaluated DOACs or VKAs versus no anticoagulation therapy. Hsu et al. [22] compared DOACs with warfarin, whereas Weir et al. [23] assessed rivaroxaban (20 mg, 15 mg, or 10 mg) versus warfarin.

Renal function distribution differed across cohorts. In the Ballegaard et al. study [21], patients were categorized according to eGFR levels, with approximately 78.9% of anticoagulated patients and 55.3% of non-anticoagulated patients presenting with eGFR 15–29 mL/min/1.73 m^2^. In contrast, Hsu et al. [22] included a population in which the majority of patients had eGFR between 15 and 29 mL/min/1.73 m^2^, with smaller proportions presenting with eGFR <15 mL/min/1.73 m^2^.

The remaining sixteen studies evaluated patients with ESRD undergoing dialysis, most commonly hemodialysis (HD), although several cohorts also included patients receiving peritoneal dialysis (PD). In the study by Harel et al. [18], for example, 93% of patients were treated with HD and 7% with PD. Similarly, in the Siontis cohort, 94.6% of patients were receiving HD and 5.4% PD [27].

Across dialysis studies, sample sizes varied substantially, ranging from relatively small cohorts such as 28–32 patients in the Wakasugi et al. study to very large administrative database cohorts such as over 25,000 patients included in the Siontis et al. study [27,34]. The majority of dialysis cohorts compared warfarin with no anticoagulation, although several studies evaluated apixaban or other DOACs compared with warfarin or alternative anticoagulation strategies.

The baseline demographic characteristics, renal function parameters, and anticoagulant regimens compared in the included studies are presented in Table 2 (Ref. [16,17,18,19,20,21,22,23,24,25,26,27,28,29,30,31,32,33,34,35]).

**Table 2.** **Patient demographics, renal function parameters and anticoagulant regimens compared in the included trials**.

Trial	OAC/none or other OAC (comp.)	Patient number (comp.)	Male (comp.)	Age (comp.)	Dialysis modality (comp.) [HD/PD]	eGFR <15 mL/min/1.73 m^2^ (comp.)	eGFR 15–29 mL/min/1.73 m^2^ (comp.)
Advanced CKD (Stage 4 and 5 CKD)							
Stanifer et al., 2020 [20]	Apixaban 5 mg (35% of pts) or 2.5 mg (65% of pts)/warfarin	136/133	52 (38.2%)/54 (40.6%)	Median 81/81	-	-	CrCl 25–30 mL/min
Ballegaard et al., 2024 [21]	DOAC or warfarin/none	1375/1833	687/1007	Median 81/79	HD 128 (9.3%)/543 (29.6%)	162 (11.8%)/277 (15.1%)	1085 (78.9%)/1013 (55.3%)
Hsu et al., 2023 [22]	DOACs/warfarin	809/202	57.3%/57.9%	Mean 84.1/78.2	HD 0.6%/2.6%	30 (3.7%)/67 (33.2%)	779 (96.3%)/135 (66.8%)
Weir et al., 2020 [23]	Rivaroxaban 20 mg (14.7% of pts) or 15 (60.1% of pts) or 10 mg (21.1% of pts)/warfarin/4.1% not documented	781/1536	38.4%/40.7%	Mean 79.9 (SD 8.2)	-	Stage V No dialysis (3.6%/3.8%) Dialysis (14.6%/15.4%)	Stage IV 81.8%/80.9%
Patients on dialysis							
Pokorney et al., 2022 [16]	Apixaban 5 mg (71% of pts) or 2.5 mg (29% of pts)/warfarin	82/72	48/50	<65 y 32/25. 65–75 y 26/32. over 75 y 24/15	HD	ESRD	-
De Vriese et al., 2021 [17]	VKA/Rivaroxaban 10 mg/Rivaroxaban 10 mg + vitamin K2	44/46/42	25/35/28	Median 80.3/79.9/79.6	HD	ESRD	-
Harel et al., 2025 [18]	Apixaban (5 or 2.5 mg) versus warfarin versus no OAC	51/52/48	77.7%	72/71	HD 93% PD 7%	ESRD	-
Reinecke et al., 2023 [19]	Apixaban (2.5 mg)/Phenprocoumon	48/49	31 (64.6%)/37 (75.5%)	Mean 74.7/74.8	HD	ESRD	-
Roh et al., 2024 [24]	DOACs/warfarin	106/319	67.9%/65.9%	(67.8 ± 9.4)/(66.2 ± 10.7)	HD 99.5% PD 0.5%	ESRD	-
Phan et al., 2019 [25]	Warfarin/none	115/361	67/208	(67.3 ± 10.8)/(62.9 ± 13.3)	PD	ESRD	-
Moore et al., 2024 [26]	Apixaban 5 mg (21 pts) or 2.5 mg (32 pts)/warfarin	53/57	29 (54.7%)/30 (52.6%)	(68.74 ± 10.28)/(63.37 ± 16.18)	HD	ESRD	-
Siontis et al., 2018 [27]	Apixaban 5 mg (1034 pts) or 2.5 mg (1317 pts)/warfarin	2351 (9.2%)/23,172 (90.8%)	13,852 (54.3%) in total. 1280 of apixaban pts were male	68.2 ± 11.9 years	HD 94.6% PD 5.4%	ESRD	-
Sánchez Soriano et al., 2018 [28]	Acenocoumarol/none	43/31	30/19	74.7/75.5	HD	ESRD	-
Yodogawa et al., 2016 [29]	Warfarin/no OAC	30/54	24 (80%)/35 (65%)	(69.5 ± 10.7)/(70.4 ± 10.2)	HD	ESRD	-
Mavrakanas et al., 2020 [30]	Apixaban 5 mg (207 pts) or 2.5 mg (257 pts) and 57 switched doses/no OAC	521/1561	281 (54%)/824 (53%)	(68 ± 11)/(68 ± 13)	HD 87%/88% PD 13%/12%	ESRD	-
Yoon et al., 2017 [31]	Warfarin vs no OAC	2921/7053	1750 (59.9%)/4056 (57.5%)	(67.8 ± 11.0)/(66.1 ± 12.6)	HD	ESRD	-
Genovesi et al., 2017 [32]	Warfarin vs no OAC	134/149	63.1%/58.8%	Median 76	HD	ESRD	-
Chan et al., 2015 [33]	Warfarin vs aspirin vs dabigatran (150 or 75 mg) vs rivaroxaban (20 or 15 mg)	8064/6018/281/244	61.2%/57.3%/59.2%/60.5%	70.6/71.7/68.4/66.9	HD	ESRD	-
Wakasugi et al., 2014 [34]	Warfarin vs no OAC	28/32	16/23	67.8/68.4	HD	ESRD	-
Shah et al., 2014 [35]	Warfarin vs no OAC	756/870	459 (61%)/533 (61%)	(75.3 ± 8.1)/(75.1 ± 8.5)	HD or PD	ESRD	-

Data are presented as Treatment/Comparator, where the specific treatment and comparator for each trial are defined in the second column. OAC, Oral Anticoagulants; Comp., Comparators; HD, Hemodialysis; PD, Peritoneal Dialysis; eGFR, estimated Glomerular Filtration Rate; CKD, Chronic Kidney Disease; mg, milligram; DOAC, Direct Oral Anticoagulants; VKA, vitamin K antagonist; pts, patients; y, years; ESRD, End-stage Renal Disease; CrCl, Creatinine Clearance.

### 3.4 Baseline Clinical Characteristics and Comorbidities

Hypertension was highly prevalent across both advanced CKD and dialysis populations. Among the studies evaluating advanced CKD patients not receiving dialysis, hypertension was reported in 53.2% to 94.6% of patients across treatment groups [21,23]. In dialysis populations, hypertension prevalence ranged from approximately 46% to 100%, depending on the cohort [25,34].

Diabetes mellitus was also common in the included populations. In the advanced CKD cohorts, diabetes prevalence ranged from 20.6% to 43.1% [20,22], whereas in dialysis studies it ranged from 20% to 83%, reflecting the high burden of metabolic disease among patients with ESRD [20,30].

A history of stroke or transient ischemic attack was reported in several cohorts and varied across studies. In the Stanifer et al. study [20], prior stroke was present in 29.4% of apixaban-treated patients and 24.8% of warfarin-treated patients. In dialysis cohorts, the prevalence of previous stroke ranged from 10% to over 30%, depending on the population studied [26,29].

Concomitant antiplatelet therapy was frequently reported in both advanced CKD and dialysis studies. Aspirin was the most commonly reported agent, with prevalence ranging from approximately 7% to over 50% across cohorts. Some studies also reported the use of platelet ADP receptor antagonists (P2Y12) inhibitors such as clopidogrel, either alone or in combination with aspirin [27,31].

Baseline bleeding history was variably reported across the included studies. In the Ballegaard cohort, previous bleeding events were reported in 12.9% of anticoagulated patients and 17.8% of patients not receiving anticoagulation [21]. In the Phan study, prior hemorrhagic stroke was present in 0.9% of patients receiving warfarin and 1.7% of those not receiving anticoagulation [25]. The De Vriese et al. [17] randomized trial reported a history of gastrointestinal bleeding in 12, 9, and 16 patients across the VKAs, rivaroxaban, and rivaroxaban plus vitamin K2 groups, respectively. Similarly, the Wakasugi et al. [34] cohort reported a history of hemorrhagic stroke in one patient receiving warfarin and none in the control group. In the Siontis cohort, baseline bleeding history prior to matching was present in approximately 9–10% of patients [27].

Thromboembolic risk was generally high across the included populations. Reported CHA_2_DS_2_-VASc scores typically ranged between 4 and 5, indicating that the majority of patients included in these studies had a high baseline risk of stroke and systemic embolism [18,21,22,27].

Baseline clinical characteristics and comorbidities across the included studies are summarized in Table 3 (Ref. [16,17,18,19,20,21,22,23,24,25,26,27,28,29,30,31,32,33,34,35]).

**Table 3.** **Baseline patient clinical characteristics and comorbidities by treatment group**.

Trial	Concomitant antiplatelet therapy (comp.)	Hypertension (comp.)	Diabetes mellitus (comp.)	History of stroke/TIA (comp.)	Previous bleeding events (comp.)	Stroke-risk score (CHA_2_DS_2_-VASc or CHADS_2_) (comp.)
Advanced CKD patients (Stage 4 and 5 CKD)						
Stanifer et al., 2020 [20]	Aspirin 44 (32.4%)/55 (41.4%)	119 (87.5%)/118 (88.7%)	28 (20.6%)/31 (23.3%)	40 (29.4%)/33 (24.8%)	-	(4.9 ± 1.39)/(4.7 ± 1.46)
Ballegaard et al., 2024 [21]	804/991	795 (57.8%)/976 (53.2%)	463 (33.7%)/576 (31.4%)	71 (5.2%)/128 (7%)	177 (12.9%)/327 (17.8%)	≤2: 272 (19.8%)/466 (25.5%) >2: 1103 (80.2%)/1367 (74.5%)
Hsu et al., 2023 [22]	290 (35.9%)/88 (43.6%)	629 (77.8%)/162 (80.2%)	273 (33.8%)/87 (43.1%)	-	203 (25.1%)/44 (21.8%)	Mean 4.5/4.5
Weir et al., 2020 [23]	50.1%/51%	94.6%/93.8%	39.2%/38.2%	-	-	Mean 4.5
Patients on dialysis						
Pokorney et al., 2022 [16]	ASA 29/32 Clopidogrel 0/3	79/67	42/47	17 (20.7%)/12 (16.7%)	18/14	Median 4
De Vriese et al., 2021 [17]	ASA 14/15/17	-	20/20/22	16/15/9	History of GI bleeding 12/9/16	Mean 4.8/4.7/4.5
Harel et al., 2025 [18]	ASA 14 (28%)/16 (31%) P2Y inhibitor 4 (8%)/1 (2%)	46 (90%)/42 (81%)	35 (69%)/33 (64%)	7 (14%)/7 (14%)	-	Mean 4
Reinecke et al., 2023 [19]	ASA 16 (33.3%)/17 (34.7%)	-	-	-	-	Mean 4.50/4.54
Roh et al., 2024 [24]	ASA 21.7%/21% Clopidogrel 15.3%/15.3%	51.9%/47.8%	35.8%/22.5%	-	8.8%/10.4%	(2.6 ± 1.5)/(2.6 ± 1.5)
Phan et al., 2019 [25]	ASA 16 (13.9%)/62 (17.2%) Clopidogrel 30 (26.1%)/121 (33.5%)	100%	83 (72.2%)/271 (75.1%)	23 (20%)/76 (21.1%)	Prior HS 1 (0.9%)/6 (1.7%)	(4.6 ± 1.6)/(4.2 ± 1.8)
Moore et al., 2024 [26]	Antiplatelet vs NSAID (90.0% vs 68.0%)	-	-	17 (32.1%)/23 (40.4%)	-	Median 2/3
Siontis et al., 2018 [27]	Antiplatelet 154 (6.6%)/1712 (7.4%)	2342 (99.6%)/23,079 (99.6%)	1773 (75.4%)/17,348 (74.9%)	Before matching 778 (33.1%)/7683 (33.2%)	Before matching 217 (9.2%)/2319 (10.0%)	Before matching median 5.27/5.24
Sánchez Soriano et al., 2018 [28]	3 (7.0%)/17 (54.84%)	36 (83.7%)/27 (87.1%)	18 (42%)/13 (42%)	-	5 (11.6%)/2 (6.5%)	4.02/3.48
Yodogawa et al., 2016 [29]	12 (40%)/29 (54%)	17 (57%)/26 (48%)	11 (37%)/23 (43%)	3 (10%)/1 (2%)	1 (3%)/4 (7%)	CHADS2 score (1.7 ± 1.1)/(1.5 ± 1.0)
Mavrakanas et al., 2020 [30]	117 (23%)/356 (23%)	100%	419 (80%)/1251 (80%)	178 (34%)/564 (36%)	254 (49%)/752 (48%)	-
Yoon et al., 2017 [31]	ASA 1302 (44.6%)/3949 (56.0%) Other antiplatelet 749 (25.6%)/2161 (30.6%)	2612 (89.4%)/5589 (79.2%)	1259 (43.1%)/2529 (35.9%)	-	-	1327 (45.4%)/2562 (36.3%)
Genovesi et al., 2017 [32]	51.7%/51.1%	82.8%/80.8%	28.7%/32.9%	15.6%/16.2%	-	2 or more 97.4%/95.8%
Chan et al., 2015 [33]	3.1%/100%/5.6%/3.4%	88.5%/88.9%/86.9%/84.9%	67.9%/66.8%/70.4%/67.8%	12.7% (1024)/14.3% (861)/12.5% (35)/16.0% (39)	Past 30 days major bleed 3.3% (266)/0.7% (42)/4.1% (12)/4.2% (10)	Median 2.4/2.4/2.3/2.2
Wakasugi et al., 2014 [34]	17 (61%)/15 (47%)	13 (46%)/16 (50%)	6 (21%)/9 (28%)	4 (14%)/8 (26%)*	HS 1 (4%)/0	CHADS2 score 2 or more: 52 patients
Shah et al., 2014 [35]	ASA 166 (22%)/241 (28%) Clopidogrel 30 (4%)/58 (7%)	582 (77%)/655 (75%)	330 (44%)/340 (39%)	42 (6%)/44 (5%)	65 (9%)/139 (16%)	CHADS2 score 2 or more: 580 (77%)/596 (69%)

Data are presented as Treatment/Comparator, where the specific treatment and comparator for each trial are defined in the second column of Table 2. Comp., Comparators; TIA, Transient Ischemic Attack; CKD, Chronic Kidney Disease; ASA, Aspirin; GI, Gastrointestinal; NSAID, Non-Steroidal Anti-Inflammatory Drug; HS, Hemorrhagic Stroke; Stroke-risk scores are CHA_2_DS_2_-VASc scores unless explicitly labeled CHADS_2_ in the cell. Both are stroke risk assessment scores for patients with atrial fibrillation; higher scores correspond to higher stroke risk. *Percentage as reported in the original publication, calculated on an available-case basis (n = 31 of 32) owing to missing data for one patient in this group.

### 3.5 Efficacy and Safety Outcomes of Anticoagulation Strategies

The efficacy and safety outcomes of the anticoagulation strategies evaluated across the included studies are presented below, stratified according to renal phenotype. Given the distinct clinical and pathophysiological characteristics of patients with advanced CKD not requiring dialysis compared with those undergoing renal replacement therapy, results are reported separately for non-dialysis stage 4–5 CKD and dialysis-dependent ESRD populations. Effect estimates for thromboembolic events, major bleeding, and all-cause mortality are detailed in the corresponding tables.

#### 3.5.1 Advanced CKD Population (CKD Stage 4–5)

Four studies evaluated thromboembolic and bleeding outcomes in patients with advanced CKD not receiving dialysis.

In the analysis by Stanifer et al. [20], apixaban was compared with warfarin in patients with creatinine clearance between 25 and 30 mL/min. The rate of ischemic stroke or systemic embolism was 2.81 vs 5.06 events per 100 person-years for apixaban and warfarin, respectively, corresponding to a hazard ratio (HR) of 0.55 (95% confidence interval [CI] 0.20–1.51). Major bleeding occurred less frequently in the apixaban group (3.78 vs 11.94 events per 100 person-years; HR 0.34, 95% CI 0.14–0.80). All-cause mortality was similar between groups (15.2 vs 15.3 events per 100 person-years; HR 1.02, 95% CI 0.64–1.67) [20].

The Ballegaard et al. [21] study compared oral anticoagulation with no anticoagulation in patients with reduced renal function. Among patients with eGFR greater than 15 mL/min/1.73 m^2^, DOAC therapy was associated with a lower risk of thromboembolic events compared with no treatment (HR 0.61, 95% CI 0.41–0.91), whereas VKA therapy was not associated with a significant difference (HR 0.87, 95% CI 0.59–1.27). Major bleeding occurred more frequently with anticoagulation, with HR of 1.74 (95% CI 1.31–2.30) for VKAs and 1.48 (95% CI 1.13–1.94) for DOACs compared with no treatment. Regarding mortality, DOAC use was associated with a lower risk of death (HR 0.71, 95% CI 0.63–0.80), while VKA therapy showed no statistically significant difference (HR 0.88, 95% CI 0.78–1.01). Subgroup analyses according to dialysis status demonstrated similar relative effects across groups [21].

In the cohort analyzed by Hsu et al. [22], DOAC therapy was compared with warfarin after adjustment using inverse probability of treatment weighting. DOAC use was associated with a lower risk of ischemic events (HR 0.42, 95% CI 0.22–0.79; *p* = 0.0067), while the risk of hemorrhagic events requiring hospitalization was similar between groups (HR 0.99, 95% CI 0.34–2.92; *p* = 0.98). No statistically significant difference in all-cause mortality was reported [22].

In the observational study by Weir et al. [23], rivaroxaban was compared with warfarin in patients with stage IV and stage V CKD. For stage IV CKD patients treated with rivaroxaban, the HR for ischemic stroke or systemic embolism was 1.35 (95% CI 0.61–2.96). In contrast, for those with stage V CKD, the HR was not estimable due to a zero-event rate in one of the treatment groups. Major bleeding rates were similar between treatment groups for both stage IV (HR 1.01, 95% CI 0.69–1.48) and stage V CKD (HR 0.65, 95% CI 0.32–1.34). Mortality outcomes were not reported in this analysis [23].

In summary, the available evidence for non-dialysis stage 4–5 CKD is limited but consistently favors DOACs, particularly apixaban, which offer thromboembolic protection similar to VKAs, with a comparable or reduced risk of major bleeding. Observational data additionally raise the possibility of a survival benefit with anticoagulation, most evident for DOACs, although this is offset by a higher absolute bleeding risk. Overall, the available evidence in this phenotype favours DOACs over VKAs, but the small number of studies and the predominance of observational, non-randomized designs preclude firm conclusions.

#### 3.5.2 Dialysis Population

Sixteen studies evaluated the efficacy and safety of anticoagulation strategies in patients with ESRD undergoing dialysis.

In the randomized trial by Pokorney et al. [16], stroke occurred in 2% of patients receiving apixaban and 3% of those receiving warfarin, with no systemic embolic events reported. Major or clinically relevant non-major bleeding occurred in 21 of 82 patients (26%) receiving apixaban and 16 of 72 patients (22%) receiving warfarin; the corresponding 1-year Kaplan–Meier incidence was 31.5% and 25.5%, respectively (HR 1.20, 95% CI 0.63–2.30). All-cause mortality occurred in 26% of apixaban-treated patients and 18% of warfarin-treated patients [16].

The randomized study by De Vriese et al. [17] compared VKAs with rivaroxaban with or without vitamin K2 supplementation. The composite outcome of fatal and non-fatal cardiovascular events occurred less frequently in the rivaroxaban groups compared with VKAs (HR 0.41, 95% CI 0.25–0.68 for rivaroxaban vs VKA; HR 0.34, 95% CI 0.19–0.61 for rivaroxaban plus vitamin K2 vs VKA). Life-threatening or major bleeding was less frequent with rivaroxaban (HR 0.39, 95% CI 0.17–0.90) compared with VKAs. All-cause mortality rates were 30.6 events per 100 person-years overall, with no statistically significant differences between treatment arms [17].

In the pilot trial by Harel et al. [18], stroke, triansient ischemic attack or systemic embolism occurred in one patient receiving apixaban and none receiving warfarin. Major bleeding occurred in two patients receiving apixaban and four patients receiving warfarin (risk ratio [RR] 0.51, 95% CI 0.10–2.66). All-cause mortality occurred in two patients receiving apixaban and nine receiving warfarin (RR 0.23, 95% CI 0.05–1.00) [18].

The Reinecke et al. study compared apixaban with phenprocoumon. Ischemic stroke occurred in 0 patients receiving apixaban and 1 patient receiving phenprocoumon, while myocardial infarction occurred in 2 vs 3 patients, respectively. Major bleeding events occurred in 10.4% and 12.2% of patients receiving apixaban and phenprocoumon, respectively. All-cause mortality occurred in 18.8% of patients receiving apixaban and 24.5% receiving phenprocoumon [19].

In the observational study by Roh et al. [24], DOAC therapy was associated with a lower risk of ischemic stroke compared with warfarin (0.18 vs 1.76 events per 100 person-years; HR 0.07, 95% CI 0.02–0.34; *p* = 0.001). Rates of gastrointestinal bleeding were numerically higher in the DOAC group (HR 1.78, 95% CI 0.96–3.27), while intracranial hemorrhage occurred in 0 vs 0.9 events per 100 person-years in the DOAC and warfarin groups, respectively [24].

Several studies compared warfarin with no anticoagulation therapy. In the Phan et al. cohort, ischemic stroke occurred in 10 vs 11 patients (adjusted HR 2.3, 95% CI 0.94–5.4). Gastrointestinal bleeding and hemorrhagic stroke were also reported but were not significantly different between groups. Mortality occurred in 32 vs 98 patients, corresponding to an adjusted HR of 0.80 (95% CI 0.53–1.2) [25].

In the Moore et al. study, ischemic stroke or transient ischemic attack occurred in 7.5% of apixaban-treated patients and 10.5% of warfarin-treated patients, while symptomatic bleeding occurred in 39.6% and 36.8%, respectively (*p* = 0.918); major bleeding occurred in 42.9% and 52.4% of these patients. All-cause mortality occurred in 50.9% and 43.9% of patients in the apixaban and warfarin groups [26].

The large observational cohort by Siontis et al. [27] reported ischemic stroke or systemic embolism in 81 apixaban-treated patients and 373 warfarin-treated patients, corresponding to an HR of 0.88 (95% CI 0.69–1.12). Major bleeding occurred less frequently with apixaban (HR 0.72, 95% CI 0.59–0.87). Mortality rates were 159 vs 753 deaths, with an HR of 0.85 (95% CI 0.71–1.01) [27].

Additional observational cohorts comparing anticoagulation (a VKA or a DOAC) with no anticoagulation or with alternative antithrombotic regimens demonstrated heterogeneous results, with inconsistent stroke reduction and variable bleeding and mortality signals. These findings are detailed in Table 4 (Ref. [16,17,18,19,20,21,22,23,24,25,26,27,28,29,30,31,32,33,34,35]).

**Table 4.** **Efficacy and safety outcomes associated with the anticoagulant strategies compared in the included studies**.

Trial	Comparators (OAC/none or other OAC)	Thromboembolism—effect estimate (95% CI), where available*	Major bleeding—effect estimate (95% CI), where available*	All-cause death—effect estimate (95% CI), where available*
Advanced CKD patients (Stage 4 and 5 CKD)				
Stanifer et al., 2020 [20]	Apixaban 5 mg (35% of pts) or 2.5 mg (65% of pts)/warfarin	IS or SE events per 100PY: (2.81/5.06) [0.55 (0.20–1.51)] MI events per 100PY: (2.34/1.49) [1.60 (0.38–6.69)]	Major bleeding ISTH events per 100PY: (3.78/11.94) [0.34 (0.14–0.80)]	Events per 100PY: 15.2/15.3 [1.02 (0.64–1.67)]
Ballegaard et al., 2024 [21]	DOAC or warfarin/none	eGFR >15. VKA vs no treatment 0.87 (0.59–1.27, *p* = 0.48). DOAC vs no treatment 0.61 (0.41–0.91, *p* = 0.02). OAC vs no OAC Dialysis 0.74 (0.36–1.50) vs non-dialysis 0.74 (0.56–0.98) *p* for interaction 0.922.	eGFR >15. VKA vs no treatment 1.74 (1.31–2.30, *p* < 0.001). DOAC vs no treatment 1.48 (1.13–1.94, *p* = 0.005). OAC vs no OAC Dialysis 1.35 (0.88–2.08) vs non-dialysis 1.45 (1.18–1.77) *p* for interaction 0.71.	eGFR >15. VKA vs no treatment 0.88 (0.78–1.01), *p* = 0.07. DOAC vs no treatment 0.71 (0.63–0.80), *p* < 0.001. OAC vs no OAC Dialysis 1.16 (0.92–1.46) vs non-dialysis 0.77 (0.70–0.84) *p* for interaction 0.001.
Hsu et al., 2023 [22]	DOACs/warfarin Results after IPTW	Any ischemia: (17/35) [0.42 (0.22–0.79), *p* = 0.0067]	Hemorrhagic events that led to hospitalization: (9/7) [0.99 (0.34–2.92), *p* = 0.98]	NR
Weir et al., 2020 [23]	Rivaroxaban 20 mg (15% of pts) or 15 mg (60% of pts) or 10 mg (20% of pts)/warfarin	IS or SE stage IV: [1.35 (0.61–2.96) *p* = 0.46] stage V: no events in one group HR = 0	Stage IV: [1.01 (0.69–1.48) *p* = 0.95] Stage V: [0.65 (0.32–1.34) *p* = 0.24]	-
Patients on dialysis				
Pokorney et al., 2022 [16]	Apixaban 5 mg (71% of pts) or 2.5 mg (29% of pts)/warfarin	Stroke 2 (2%)/2 (3%) SE 0/0 [21]	ISTH major bleed 9 (11%)/7 (10%) Major or clinically relevant nonmajor bleeding 21 (26%)/16 (22%) 1-year Kaplan–Meier rates 31.5% and 25.5% [HR 1.20 (95% CI 0.63–2.30)]	21 (26%)/13 (18%)
De Vriese et al., 2021 [17]	VKA/Rivaroxaban 10 mg/Rivaroxaban 10 mg + vitamin K2	Fatal and nonfatal CV events Rivaroxaban vs VKA [0.41 (95% CI, 0.25 to 0.68; *p* = 0.0006)] Rivaroxaban + Vitamin K2 vs VKA [0.34 (95% CI, 0.19 to 0.61; *p* = 0.0003)]	Major bleeding ISTH Rivaroxaban vs VKA: HR 0.39 (95% CI, 0.17 to 0.90; *p* = 0.03) Rivaroxaban + Vitamin K2 vs VKA: HR 0.48 (95% CI, 0.22 to 1.08; *p* = 0.08)	The rate of all–cause death was 30.6 per 100PY, with 33.7 per 100PY in the VKA group, 28.3 per 100PY in the rivaroxaban group, 30.2 per 100PY in the rivaroxaban and vitamin K2 group (*p* = 0.66 for difference between the three arms)
Harel et al., 2025 [18]	Apixaban (5 or 2.5 mg) vs warfarin vs no OAC	Stroke, TIA or SE: 1/0 [RR 3.06, 95% CI (0.13–73.36), *p* > 0.05]	Major bleeding ISTH: 2/4 [RR 0.51, 95% CI (0.10–2.66), *p* > 0.05]	All-cause mortality: 2/9 [RR 0.23, 95% 0.05–1.00, *p* = 0.05]
Reinecke et al., 2023 [19]	Apixaban (2.5 mg)/Phenprocoumon	IS: 0/1 MI: 2/3	Major bleeding ISTH 5 (10.4%) 6 (12.2%), *p* = 1.0	All-cause mortality 9 (18.8%)/12 (24.5%), *p* = 0.7820
Roh et al., 2024 [24]	DOACs/warfarin Results after IPTW	IS events per 100PY: (0.18/1.76) [0.07 (0.02–0.34), *p* = 0.001] MI events per 100PY: (1.86/1.23) [1.32 (0.68–2.58), *p* = 0.41]	ICH events per 100PY: (0/0.9) GI bleeding events per 100PY: (2.65/1.31) [1.78 (0.96–3.27)] *p* = 0.06	-
Phan et al., 2019 [25]	Warfarin/none	IS 10/11 [2.3 (0.94–5.4) *p* = 0.07]	HS 2/3 [2.0 (0.32–12.8) *p* = 0.46] GI bleed 7/22 [0.92 (0.39–2.2) *p* = 0.86]	32/98 [0.80 (0.53–1.2) *p* = 0.28 adjusted]
Moore et al., 2024 [26]	Apixaban (5 or 2.5 mg)/warfarin	IS or TIA: 4 (7.5%)/6 (10.5%), *p* = 0.742	Symptomatic bleeding 21 (39.6%)/21 (36.8%), *p *= 0.918 Major bleeding ISTH 9 (42.9%)/11 (52.4%) (% of patients with symptomatic bleeding)	27 (50.9%)/25 (43.9%)
Siontis et al., 2018 [27]	Apixaban (5 or 2.5 mg)/warfarin	IS or SE: 81/373 [0.88 (0.69–1.12), *p* = 0.29]	Major bleeding events 129/715. Event rate per 100PY: 19.7/22.9 [0.72 (0.59–0.87), *p* < 0.001]	159/753 [0.85 (0.71–1.01) *p* = 0.06]
Sánchez Soriano et al., 2018 [28]	Acenocoumarol/none	6/1 IS	Severe bleeding 13/3 Fatal bleeding 6/0	Acenocoumarol was not associated with lower mortality (2.78 vs 2.01 per 10PY, *p* = 0.304). In the multivariate analysis adjusting for Propensity Index (PS) and CHA2DS2–VASc, anticoagulation had a neutral effect on survival [HR = 1, 0.76, 95% CI (0.35–1.66), *p* = 0.494]
Yodogawa et al., 2016 [29]	Warfarin/no OAC	Warfarin not associated with risk for stroke [HR 1.07; 95% CI (0.20–5.74), *p* > 0.05]	4 pts were admitted to the hospital for ICH or GI bleeding The Kaplan–Meier analysis for “bleeding–free survival” showed no statistically significant difference between the groups (HR and CI NS)	21 patients died in total. The Kaplan–Meier analysis showed no statistically significant difference in overall survival between the warfarin and non–warfarin groups (HR and CI NR)
Mavrakanas et al., 2020 [30]	Apixaban (5 or 2.5 mg)/no OAC	Any stroke, TIA or embolism Standard dose: 13.6/5.6, [2.24 (1.03 to 4.86) *p* = 0.04] Reduced dose: 5.7/6.1 [1.11 (0.43 to 2.85) *p* = 0.84]	Fatal or intracranial bleeding Standard dose 9.8/1.7, [4.61 (1.91 to 11.15) *p* = 0.001] Reduced dose: 2.9/1.4, [2.02 (0.58 to 7.04) *p* = 0.27]	Apixaban use was associated with lower all–cause mortality rates compared with no anticoagulation (HR, 0.58; 95% CI, 0.43 to 0.78).
Yoon et al., 2017 [31]	Warfarin vs no OAC	MI 265 (9.6%)/259 (9.3%) *p* = 0.783 IS 204 (7.4%)/201 (7.2%) *p* = 0.877	HS 84 (3.0%)/50 (1.8%) *p* = 0.003	-
Genovesi et al., 2017 [32]	Warfarin vs no OAC	25 events in total 0.44 (0.16–1.20) *p* = 0.1	55 events in total 1.16 (0.48–2.82) *p* = 0.7	75/95 0.91 [0.56–1.48] *p* = 0.7
Chan et al., 2015 [33]	Warfarin vs ASA vs dabigatran (150 or 75 mg) vs rivaroxaban (20 or 15 mg)	IS or arterial embolism 244/168/13/8. Dabigatran 0.81 (0.66–0.99) Warfarin 1.71 (0.97–2.99) ASA 1.80 (0.89–3.64)	HS 121/75/1/0 ASA vs warfarin 0.74 (0.55–0.98) Dabigatran vs warfarin 0.26 (0.04–1.84)	-
Wakasugi et al., 2014 [34]	Warfarin vs no OAC	IS 8/5 [1.94 (0.63–5.93)]	3/4 [0.85 (0.19–3.64)]	9/9 [1.00 (0.40–2.52)]
Shah et al., 2014 [35]	Warfarin vs no OAC	IS 52/55 [1.14 (0.78–1.67)]	Hospital admission or ED visit for ICH, GI bleeding, hematuria: 149/126 [1.44 (1.13–1.85)]	-

Data for clinical outcomes are presented as Treatment/Comparator, where the specific treatment and comparator for each trial are defined in the second column. CKD, Chronic Kidney Disease; pts, patients; mg, milligram; IS, Ischemic Stroke; SE, Systemic Embolism; 100PY, 100 person-years; MI, Myocardial Infarction; ISTH, International Society on Thrombosis and Haemostasis; DOAC, Direct Oral Anticoagulant; eGFR, estimated Glomerular Filtration Rate; VKA, Vitamin K Antagonist; OAC, Oral Anticoagulant; IPTW, Inverse Probability of Treatment Weighting; CV, Cardiovascular; ICH, Intracranial Hemorrhage; HS, Hemorrhagic Stroke; GI, Gastrointestinal; TIA, Transient Ischemic Attack; NR; Not Reported; ASA, Aspirin; ED, Emergency Department. *Data are presented as reported by each original study and may comprise raw event counts (n (%) or n/N), crude event rates (events per 100 person-years), and, where available, effect estimates with 95% confidence intervals. Effect estimates are hazard ratios (HR) unless explicitly labeled as risk ratios (RR). Raw event counts and event rates are descriptive data and should not be interpreted as effect estimates.

In summary, the evidence in dialysis-dependent ESRD is markedly more heterogeneous and less conclusive. Comparisons of warfarin with no anticoagulation yielded inconsistent effects on stroke, with several cohorts showing no significant reduction alongside a frequently higher bleeding risk, so that a clear net clinical benefit of warfarin could not be established. Where DOACs were compared with VKAs, apixaban was generally associated with a similar risk of stroke or systemic embolism and a lower risk of major bleeding; however, the randomized trials were small and underpowered. Taken together, the data suggest that, when anticoagulation is pursued in this population, a factor Xa inhibitor is preferable to a VKA on safety grounds, whereas the more fundamental question of whether to anticoagulate at all in dialysis-dependent AF remains unresolved.

#### 3.5.3 Quantitative Synthesis (Dialysis Randomized Trials)

Four randomized trials comparing factor Xa inhibitors with VKAs in dialysis-dependent patients (n = 486) were eligible for quantitative synthesis (Fig. 2, Ref. [17,18,19]). Factor Xa inhibitors were associated with a statistically significant reduction in ISTH major bleeding compared with VKAs (pooled RR 0.64, 95% CI 0.42–0.99; I^2^ = 0%). In sensitivity analyses using the Mantel–Haenszel method and the Hartung–Knapp small-sample adjustment, this association was attenuated and no longer reached statistical significance (RR 0.67, 95% CI 0.44–1.03 and RR 0.64, 95% CI 0.34–1.21, respectively). A reduction in stroke, transient ischemic attack or systemic embolism did not reach statistical significance (RR 0.46, 95% CI 0.20–1.02; I^2^ = 0%), and no difference was observed for all-cause mortality (RR 0.88, 95% CI 0.58–1.35; I^2^ = 46%). In a post-hoc sensitivity analysis restricted to the two trials judged to be at low risk of bias, the direction and magnitude of all three pooled estimates were preserved (ISTH major bleeding RR 0.56, 95% CI 0.34–0.92; stroke or systemic embolism RR 0.33, 95% CI 0.13–0.84; all-cause mortality RR 0.88, 95% CI 0.70–1.10), although based on only two trials, these estimates should be regarded as exploratory rather than confirmatory [17,19]. These pooled estimates were concordant with the recently published randomized-trial meta-analysis by Kaisaier et al. [36].

**Fig. 2.**
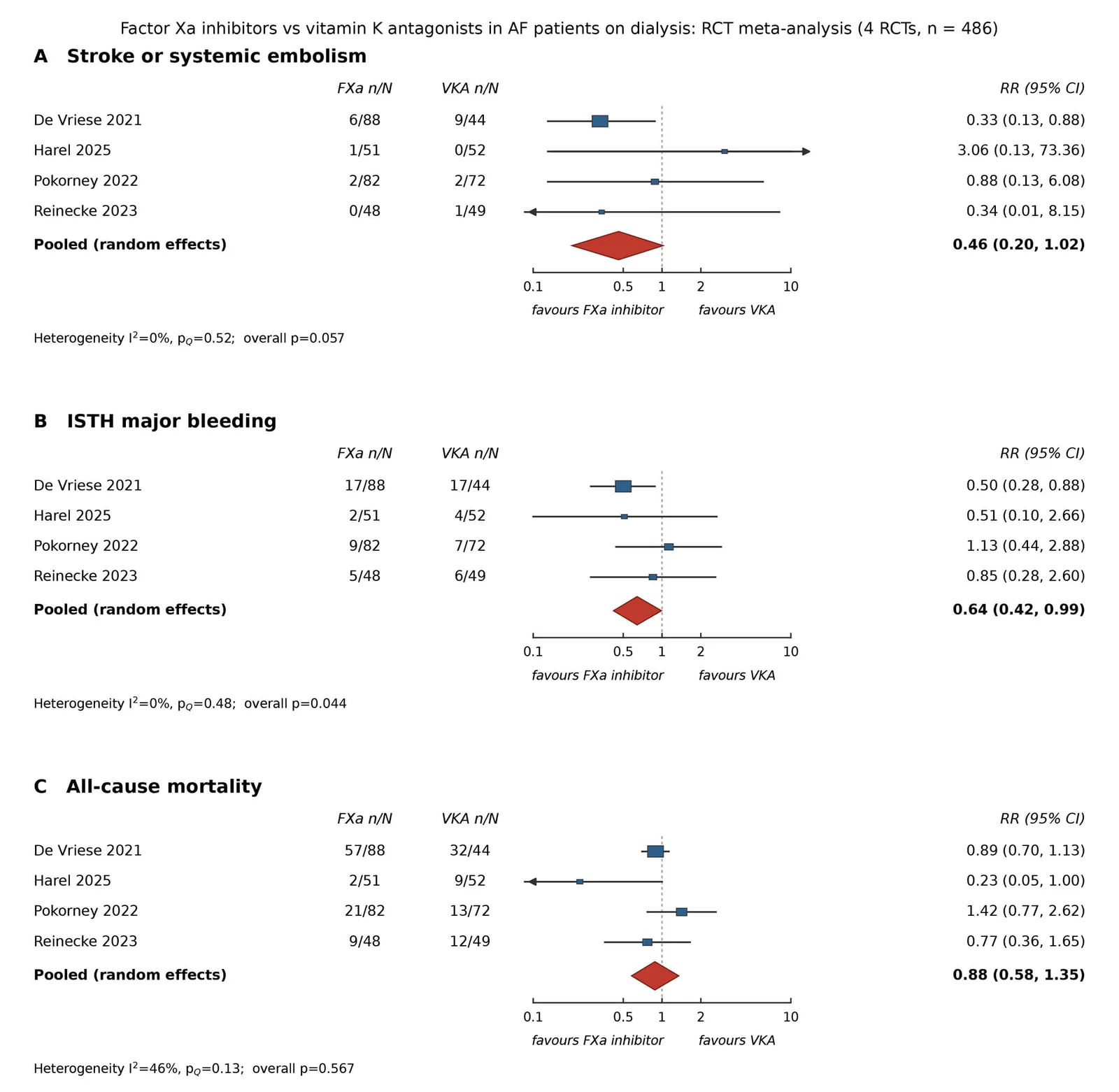
**Forest plots of efficacy and safety outcomes in the meta-analysis of randomized controlled trials comparing factor Xa inhibitors with VKAs in patients with AF receiving dialysis**. Four randomized controlled trials, n = 486. (A) stroke, transient ischaemic attack, or systemic embolism, (B) ISTH major bleeding, (C) all-cause mortality. Risk ratios (RR) with 95% confidence intervals (CI) were pooled using an inverse-variance random-effects model with a 0.5 continuity correction for zero-event cells. n/N, actual number of participants experiencing the outcome / total number of randomized participants in the corresponding study arm (crude intention-to-treat counts as reported in the source publications). For De Vriese et al. [17], the combined 'life-threatening or major bleeding' outcome was extracted (VKA 17/44; pooled rivaroxaban arms 17/88). That trial defines life-threatening and major bleeding as mutually exclusive rather than nested categories, so its 'major bleeding' row excludes fatal and symptomatic intracranial bleeding. Since fatal and critical-organ bleeding are constitutive of ISTH major bleeding, the combined row is the closest available approximation to the outcome reported in the other three trials. The thromboembolic outcome is a composite of adjudicated stroke (ischaemic or haemorrhagic), transient ischaemic attack, and systemic embolism, as prespecified and as reported by the included trials. Harel et al. [18] randomised patients to apixaban, warfarin or no anticoagulation; only the apixaban and warfarin arms enter this comparison. Within those two arms there were no strokes, and the single event contributed is the transient ischaemic attack reported in the apixaban arm; the one adjudicated stroke in that trial occurred in the no-anticoagulation arm. Reinecke et al. [19] report ischaemic stroke and transient ischaemic attack as a single combined category. RCT, randomized controlled trial; CI, confidence interval; FXa, factor Xa; ISTH, International Society on Thrombosis and Haemostasis; RR, risk ratio; VKA, vitamin K antagonist.

It should be noted that the analysis by Kaisaier et al. [36] was restricted exclusively to randomized trials of factor Xa inhibitors versus VKAs in dialysis-dependent patients. The present review differs in scope: it additionally encompasses patients with non-dialysis stage 4–5 CKD, incorporates oral anticoagulant–versus–no-anticoagulation comparisons, and integrates the larger body of observational evidence, thereby characterising the differential risk–benefit profile of oral anticoagulation across the entire CKD spectrum rather than within the dialysis randomized-trial subset alone. The quantitative synthesis presented here therefore serves to corroborate, within this broader qualitative framework, the safety signal favouring factor Xa inhibitors observed in that focused analysis, while the principal contribution of the present work remains the stratified appraisal of evidence across renal phenotypes.

### 3.6 Risk of Bias Due to Missing Results

The risk of bias arising from missing results was evaluated by cross-referencing published reports with original trial registries and study protocols, where such records were available. This assessment focused on identifying potential selective non-reporting of critical endpoints. Among the studies with accessible pre-registered protocols, no significant discrepancies were identified between the intended methods and final reported results. Due to the inherent clinical and methodological heterogeneity across the 20 included studies, statistical assessment via funnel plots was deemed inappropriate.

## 4. Discussion

This systematic review summarizes the available evidence regarding the efficacy and safety of oral anticoagulation in patients with AF and advanced CKD, including both patients with stage 4–5 CKD not requiring dialysis and those with ESRD undergoing dialysis. A total of twenty studies were included, encompassing randomized trials, prospective cohorts, and predominantly retrospective observational studies. Overall, the evidence base is characterized by marked heterogeneity in study design, patient selection, anticoagulant regimens, and outcome definitions, limiting direct cross-study comparability.

One of the most important observations emerging from the present review is the marked difference in the available evidence between patients with advanced CKD not receiving dialysis and those undergoing dialysis therapy. Only a limited number of studies evaluated anticoagulation in patients with stage 4–5 CKD not requiring dialysis, whereas the majority of the literature focused on dialysis populations. This imbalance reflects the persistent clinical uncertainty in dialysis populations, where competing thromboembolic and bleeding risks are both markedly amplified.

In patients with advanced CKD not requiring dialysis, the available studies generally suggest that oral anticoagulation may reduce thromboembolic risk, particularly when DOACs are used. In the analysis by Stanifer et al. [20], apixaban was associated with lower rates of major bleeding compared with warfarin while maintaining comparable rates of thromboembolic events and mortality. Similarly, in the observational analysis by Hsu et al. [22], DOAC therapy was associated with a lower incidence of ischemic events compared with warfarin, without a significant difference in hemorrhagic complications. The Ballegaard cohort further suggested that DOAC therapy may reduce thromboembolic events and mortality compared with no anticoagulation, although this benefit was accompanied by an increased risk of major bleeding [21]. Taken together, these findings suggest that stage 4–5 CKD not requiring dialysis may represent a therapeutic window in which DOAC therapy offers a more favorable safety profile than VKAs while preserving thromboembolic protection. However, the strength of this conclusion remains limited by the predominance of observational data.

The evidence is more complex and less consistent in patients with ESRD undergoing dialysis. Across the dialysis studies included in this review, anticoagulation strategies varied widely, including comparisons between warfarin and no anticoagulation, DOACs and warfarin, or alternative anticoagulation regimens. In many observational cohorts comparing warfarin with no anticoagulation, stroke reduction was inconsistent, and several studies demonstrated either neutral findings or an unfavorable balance between thromboembolic prevention and bleeding risk. For example, in the cohorts by Shah et al., Genovesi et al., and Yodogawa et al., warfarin was not clearly associated with a reduction in stroke risk, while bleeding complications were either increased or not significantly different compared with no anticoagulation [29,32,35].

DOACs have emerged as a potential alternative to VKAs in dialysis populations, although the evidence remains limited. The large observational cohort by Siontis et al. [27] demonstrated lower rates of major bleeding with apixaban compared with warfarin, while thromboembolic rates were similar between treatment groups. Additional observational studies, such as those by Roh and Moore, also suggested comparable or lower thromboembolic risk with DOAC therapy compared with warfarin [24,26]. However, randomized evidence in dialysis populations remains scarce. The available randomized trials in dialysis-dependent patients, including the RENAL-AF trial by Pokorney et al. [16], the Valkyrie trial by De Vriese et al. [17], the pilot trial by Harel et al. [18], and the AXADIA-AFNET 8 trial by Reinecke et al. [19], provided important randomized evidence, but each was individually limited by small sample sizes and therefore had limited power to detect modest differences in clinical outcomes. This limitation at the individual trial level provided the rationale for pooling these trials in a quantitative synthesis, which aimed to provide more precise estimates than any single study could achieve alone. Consequently, while apixaban appears to demonstrate a more favorable bleeding profile compared with warfarin, definitive evidence of net clinical benefit in dialysis-dependent ESRD remains lacking.

Bleeding risk remains a major concern in patients with advanced CKD, particularly in dialysis populations. Across the included studies, major bleeding events were frequently reported and were often more common among patients receiving anticoagulation. The heightened bleeding risk likely reflects uremia-associated platelet dysfunction, endothelial abnormalities, altered coagulation pathways, vascular access interventions, and the frequent concomitant use of antiplatelet agents. In dialysis patients, procedural anticoagulation and repeated vascular manipulation may further compound hemorrhagic vulnerability [16,26,27].

The baseline characteristics summarized in Tables 2,3 further illustrate the complexity of anticoagulation management in this population. Patients included in the reviewed studies were generally elderly and had a high burden of cardiovascular comorbidities, including hypertension, diabetes mellitus, and prior stroke. Moreover, CHA_2_DS_2_-VASc scores were typically high, indicating substantial baseline thromboembolic risk. At the same time, many patients had a history of previous bleeding events and were receiving concomitant antiplatelet therapy, further increasing the complexity of risk stratification. These competing risks underscore the need for individualized clinical decision-making rather than uniform anticoagulation strategies [17,19,20,21].

Another important consideration is the methodological heterogeneity of the available evidence. Most studies included in this review were retrospective observational cohorts, which are inherently subject to confounding and selection bias. Although several studies attempted to address these limitations through propensity score matching or inverse probability of treatment weighting, residual confounding cannot be excluded. In addition, differences in outcome definitions, anticoagulant dosing strategies, and follow-up durations complicate direct comparisons between studies.

The scarcity of adequately powered RCTs in dialysis populations remains a critical evidence gap. Randomized trials evaluating anticoagulation strategies in dialysis populations remain challenging to conduct due to difficulties in recruitment and high competing risks of mortality. Nevertheless, further randomized evidence is necessary to clarify the optimal anticoagulation strategy in patients with ESRD and AF.

Our findings are broadly consistent with prior systematic reviews and meta-analyses evaluating anticoagulation in advanced CKD and ESRD, which have similarly reported uncertain stroke reduction with warfarin in dialysis populations and a potential safety advantage of apixaban over VKAs. Several previous reviews have concluded that warfarin does not consistently reduce ischemic stroke in dialysis-dependent patients and may increase major bleeding risk, while DOACs—particularly apixaban—appear associated with lower bleeding rates. However, most prior analyses were limited by reliance on retrospective cohorts and heterogeneity in patient populations. The present review extends these observations by separately analyzing non-dialysis stage 4–5 CKD and dialysis-dependent ESRD, highlighting a differential risk–benefit profile across the CKD spectrum [36,37,38,39].

When the randomized evidence in dialysis-dependent patients was pooled quantitatively, factor Xa inhibitors were associated with a significant reduction in major bleeding compared with VKAs (pooled risk ratio [RR] 0.64, 95% confidence interval [CI] 0.42–0.99), although this association did not persist in Mantel–Haenszel or Hartung–Knapp sensitivity analyses, whereas the estimates for stroke, transient ischemic attack or systemic embolism (pooled RR 0.46, 95% CI 0.20–1.02) and all-cause mortality (RR 0.88, 95% CI 0.58–1.35) were neutral and imprecise, with CIs crossing unity. This pattern—a measurable safety advantage without a demonstrable efficacy or survival benefit—mirrors the recent randomized-trial meta-analysis by Kaisaier et al. [36] and reinforces the interpretation that the available trials remain underpowered to establish the net clinical benefit of anticoagulation in this population. It nonetheless supports a factor Xa inhibitor, and apixaban in particular, as the preferred agent over a VKA when anticoagulation is pursued in dialysis-dependent AF, while leaving the more fundamental question of whether to anticoagulate at all unresolved.

Despite these limitations, this review synthesizes the contemporary evidence base evaluating anticoagulation in advanced CKD across distinct renal phenotypes. The findings highlight the delicate balance between thromboembolic prevention and bleeding risk in this population and underscore the need for individualized treatment decisions based on patient-specific risk profiles.

## 5. Limitations

Several limitations should be acknowledged. First, the majority of included studies were retrospective observational cohorts, introducing potential residual confounding and treatment selection bias despite statistical adjustment methods. Second, substantial heterogeneity existed in outcome definitions, anticoagulant dosing strategies, dialysis modality distribution, and follow-up duration, precluding quantitative meta-analysis of the overall evidence base. Third, RCTs in dialysis populations were limited in number and sample size, restricting definitive conclusions regarding net clinical benefit. Fourth, important modifiers such as time in therapeutic range for warfarin, appropriateness of DOACs dose adjustment, prior bleeding history, frailty status, and adherence were inconsistently reported. Moreover, the quantitative synthesis was restricted to four small randomized trials of factor Xa inhibitors versus VKAs in dialysis-dependent patients (n = 486); sparse event numbers produced wide confidence intervals for stroke and mortality, phenprocoumon and warfarin were pooled as a single VKA category, the two rivaroxaban arms of the De Vriese trial were combined, and no randomized comparison against no anticoagulation was available. In addition, the composite thromboembolic outcome included transient ischemic attacks where these were reported, but De Vriese et al. [17] and Pokorney et al. [16] did not report transient ischemic attacks separately, so such events could not contribute from those trials; the composite is therefore applied asymmetrically across the included studies. The small number of studies within each renal stratum, together with heterogeneous comparators and dosing regimens, precluded a formal meta-regression across the CKD spectrum; such an analysis would have been underpowered and prone to ecological bias, and remains a direction for future work as further randomized data accrue. Finally, publication bias cannot be excluded, particularly given the reliance on published English-language studies.

## 6. Conclusion

The available evidence regarding oral anticoagulation in patients with AF and advanced CKD remains heterogeneous and limited. In patients with stage 4–5 CKD not requiring dialysis, the available studies suggest that DOACs may provide comparable or improved thromboembolic protection with a potentially lower risk of major bleeding compared with VKAs. However, the number of studies in this population remains relatively small, and most evidence derives from observational analyses or subgroup evaluations of larger trials.

In contrast, the evidence in patients undergoing dialysis is less consistent. Observational studies comparing warfarin with no anticoagulation have reported mixed findings regarding thromboembolic prevention, while several cohorts suggest an increased risk of bleeding complications associated with anticoagulation therapy. Studies evaluating DOACs in dialysis populations have generally demonstrated similar or lower bleeding risk compared with VKAs, although randomized data remain limited.

Overall, patients with advanced CKD represent a population with a high burden of both thromboembolic and bleeding risk, making anticoagulation decisions particularly complex. The current literature is characterized by substantial methodological heterogeneity, a predominance of retrospective observational studies, and limited randomized evidence.

Further adequately powered randomized controlled trials are needed to clarify the optimal anticoagulation strategy in patients with advanced CKD, particularly among those receiving dialysis. Until more robust evidence becomes available, anticoagulation decisions in this population should remain individualized, taking into account the balance between thromboembolic risk, bleeding risk, and patient-specific clinical characteristics.

## Data Availability

All data relevant to the study are included in the article or uploaded as supplementary information.

## References

[1] Linz D, Gawalko M, Betz K, Hendriks JM, Lip GYH, Vinter N (2024). Atrial fibrillation: epidemiology, screening and digital health. The Lancet Regional Health. Europe.

[2] Staerk L, Wang B, Preis SR, Larson MG, Lubitz SA, Ellinor PT (2018). Lifetime risk of atrial fibrillation according to optimal, borderline, or elevated levels of risk factors: cohort study based on longitudinal data from the Framingham Heart Study. BMJ (Clinical Research Ed.).

[3] Kim SM, Jeong Y, Kim YL, Kang M, Kang E, Ryu H (2023). Association of Chronic Kidney Disease With Atrial Fibrillation in the General Adult Population: A Nationwide Population-Based Study. Journal of the American Heart Association.

[4] Navarro-Garcia JA, Keefe JA, Song J, Li N, Wehrens XHT (2025). Mechanisms underlying atrial fibrillation in chronic kidney disease. Journal of Molecular and Cellular Cardiology.

[5] Goette A, Kalman JM, Aguinaga L, Akar J, Cabrera JA, Chen SA (2017). EHRA/HRS/APHRS/SOLAECE expert consensus on atrial cardiomyopathies: Definition, characterization, and clinical implication. Heart Rhythm.

[6] Lutz J, Menke J, Sollinger D, Schinzel H, Thürmel K (2014). Haemostasis in chronic kidney disease. Nephrology, Dialysis, Transplantation: Official Publication of the European Dialysis and Transplant Association - European Renal Association.

[7] Boccardo P, Remuzzi G, Galbusera M (2004). Platelet dysfunction in renal failure. Seminars in Thrombosis and Hemostasis.

[8] Baaten CCFMJ, Vondenhoff S, Noels H (2023). Endothelial Cell Dysfunction and Increased Cardiovascular Risk in Patients With Chronic Kidney Disease. Circulation Research.

[9] Parul F, Ratnani T, Subramani S, Bhatia H, Ashmawy RE, Nair N (2025). Anticoagulation in Patients with End-Stage Renal Disease: A Critical Review. Healthcare (Basel, Switzerland).

[10] Ruff CT, Giugliano RP, Braunwald E, Hoffman EB, Deenadayalu N, Ezekowitz MD (2014). Comparison of the efficacy and safety of new oral anticoagulants with warfarin in patients with atrial fibrillation: a meta-analysis of randomised trials. Lancet (London, England).

[11] Klil-Drori AJ, Tagalakis V (2018). Direct Oral Anticoagulants in End-Stage Renal Disease. Seminars in Thrombosis and Hemostasis.

[12] Van Gelder IC, Rienstra M, Bunting KV, Casado-Arroyo R, Caso V, Crijns HJGM (2024). 2024 ESC Guidelines for the management of atrial fibrillation developed in collaboration with the European Association for Cardio-Thoracic Surgery (EACTS). European Heart Journal.

[13] Joglar JA, Chung MK, Armbruster AL, Benjamin EJ, Chyou JY, Cronin EM (2024). 2023 ACC/AHA/ACCP/HRS Guideline for the Diagnosis and Management of Atrial Fibrillation: A Report of the American College of Cardiology/American Heart Association Joint Committee on Clinical Practice Guidelines. Circulation.

[14] Sterne JAC, Savović J, Page MJ, Elbers RG, Blencowe NS, Boutron I (2019). RoB 2: a revised tool for assessing risk of bias in randomised trials. BMJ (Clinical Research Ed.).

[15] Gualdi-Russo E, Zaccagni L (2026). The Newcastle–Ottawa Scale for Assessing the Quality of Studies in Systematic Reviews. Publications.

[16] Pokorney SD, Chertow GM, Al-Khalidi HR, Gallup D, Dignacco P, Mussina K (2022). Apixaban for Patients With Atrial Fibrillation on Hemodialysis: A Multicenter Randomized Controlled Trial. Circulation.

[17] De Vriese AS, Caluwé R, Van Der Meersch H, De Boeck K, De Bacquer D (2021). Safety and Efficacy of Vitamin K Antagonists versus Rivaroxaban in Hemodialysis Patients with Atrial Fibrillation: A Multicenter Randomized Controlled Trial. Journal of the American Society of Nephrology: JASN.

[18] Harel Z, Smyth B, Badve SV, Blum D, Beaubien-Souligny W, Silver SA (2025). Anticoagulation for Patients with Atrial Fibrillation Receiving Dialysis: A Pilot Randomized Controlled Trial. Journal of the American Society of Nephrology: JASN.

[19] Reinecke H, Engelbertz C, Bauersachs R, Breithardt G, Echterhoff HH, Gerß J (2023). A Randomized Controlled Trial Comparing Apixaban With the Vitamin K Antagonist Phenprocoumon in Patients on Chronic Hemodialysis: The AXADIA-AFNET 8 Study. Circulation.

[20] Stanifer JW, Pokorney SD, Chertow GM, Hohnloser SH, Wojdyla DM, Garonzik S (2020). Apixaban Versus Warfarin in Patients With Atrial Fibrillation and Advanced Chronic Kidney Disease. Circulation.

[21] Ballegaard ELF, Olesen JB, Kamper AL, Feldt-Rasmussen B, Gislason G, Torp-Pedersen C (2024). Oral anticoagulation for stroke prevention in atrial fibrillation and advanced kidney disease. Research and Practice in Thrombosis and Haemostasis.

[22] Hsu CC, Chen CC, Chou CY, Chen KH, Wang SF, Chang SL (2023). Effectiveness and safety of direct oral anticoagulants versus warfarin in patients with atrial fibrillation and advanced kidney disease. Journal of Thrombosis and Thrombolysis.

[23] Weir MR, Ashton V, Moore KT, Shrivastava S, Peterson ED, Ammann EM (2020). Rivaroxaban versus warfarin in patients with nonvalvular atrial fibrillation and stage IV-V chronic kidney disease. American Heart Journal.

[24] Roh JH, Kim YG, Kim D, Park S, Park KS, Kim M (2024). Direct oral anticoagulants for oral anticoagulants-naïve Asian patients with atrial fibrillation and end-stage renal disease undergoing dialysis. Scientific Reports.

[25] Phan D, Yang SJ, Shen AYJ, Lee MS (2019). Effect of Warfarin on Ischemic Stroke, Bleeding, and Mortality in Patients with Atrial Fibrillation Receiving Peritoneal Dialysis. American Journal of Cardiovascular Drugs: Drugs, Devices, and other Interventions.

[26] Moore M, Vizcaino K, Ewing JA, St Ville M (2024). Efficacy and safety of apixaban compared to warfarin for nonvalvular atrial fibrillation in end-stage renal disease on hemodialysis. Journal of the American Pharmacists Association: JAPhA.

[27] Siontis KC, Zhang X, Eckard A, Bhave N, Schaubel DE, He K (2018). Outcomes Associated With Apixaban Use in Patients With End-Stage Kidney Disease and Atrial Fibrillation in the United States. Circulation.

[28] Sánchez Soriano RM, Albero Molina MD, Chamorro Fernández CI, Juliá-Sanchís R, López Menchero R, Del Pozo Fernández C (2018). Long-term prognostic impact of anticoagulation on patients with atrial fibrillation undergoing hemodialysis. Nefrologia.

[29] Yodogawa K, Mii A, Fukui M, Iwasaki YK, Hayashi M, Kaneko T (2016). Warfarin use and incidence of stroke in Japanese hemodialysis patients with atrial fibrillation. Heart and Vessels.

[30] Mavrakanas TA, Garlo K, Charytan DM (2020). Apixaban versus No Anticoagulation in Patients Undergoing Long-Term Dialysis with Incident Atrial Fibrillation. Clinical Journal of the American Society of Nephrology: CJASN.

[31] Yoon CY, Noh J, Jhee JH, Chang TI, Kang EW, Kee YK (2017). Warfarin Use in Patients With Atrial Fibrillation Undergoing Hemodialysis: A Nationwide Population-Based Study. Stroke.

[32] Genovesi S, Rebora P, Gallieni M, Stella A, Badiali F, Conte F (2017). Effect of oral anticoagulant therapy on mortality in end-stage renal disease patients with atrial fibrillation: a prospective study. Journal of Nephrology.

[33] Chan KE, Edelman ER, Wenger JB, Thadhani RI, Maddux FW (2015). Dabigatran and rivaroxaban use in atrial fibrillation patients on hemodialysis. Circulation.

[34] Wakasugi M, Kazama JJ, Tokumoto A, Suzuki K, Kageyama S, Ohya K (2014). Association between warfarin use and incidence of ischemic stroke in Japanese hemodialysis patients with chronic sustained atrial fibrillation: a prospective cohort study. Clinical and Experimental Nephrology.

[35] Shah M, Avgil Tsadok M, Jackevicius CA, Essebag V, Eisenberg MJ, Rahme E (2014). Warfarin use and the risk for stroke and bleeding in patients with atrial fibrillation undergoing dialysis. Circulation.

[36] Kaisaier W, Chen Y, Lip GYH, Liu C, Zhu W (2026). Safety and Efficacy of Factor Xa Inhibitors in Atrial Fibrillation Patients on Dialysis: Evidence from Four Randomized Controlled Trials. Thrombosis and Haemostasis.

[37] Rhee TM, Lee SR, Choi EK, Oh S, Lip GYH (2022). Efficacy and Safety of Oral Anticoagulants for Atrial Fibrillation Patients With Chronic Kidney Disease: A Systematic Review and Meta-Analysis. Frontiers in Cardiovascular Medicine.

[38] de Oliveira HM, Barros LP, de Campos MCAV, Daher RF, Gonçalves GB, Sequeira MT (2025). Anticoagulation Strategies for Atrial Fibrillation in CKD Stage G5 and Dialysis Patients: An Updated Scoping Review. Reviews in Cardiovascular Medicine.

[39] Parker K, Hartemink J, Saha A, Mitra R, Lewis P, Power A (2022). A systematic review of the efficacy and safety of anticoagulants in advanced chronic kidney disease. Journal of Nephrology.

