# Supplementary material for: Oral Anticoagulants in Patients With Atrial Fibrillation and Advanced or End-Stage Renal Disease: A Systematic Review and Meta-Analysis
Source: Rev Cardiovasc Med. 2026 Sep 16;27(9):52237. doi: 10.31083/RCM52237 (PMC13653193; doi:10.31083/RCM52237)
Supplement: Supplementary file 1 [file 2153-8174-27-9-52237-s1.zip › PRISMA_2020_checklist.docx]

| **Section and Topic** | **Item #** | **Checklist item** | **Location where item is reported** |
| --- | --- | --- | --- |
| **TITLE** | | |  |
| Title | 1 | Identify the report as a systematic review. | Page 1 (Title) |
| **ABSTRACT** | | |  |
| Abstract | 2 | See the PRISMA 2020 for Abstracts checklist. | Page 1. The abstract includes background, methods, results, conclusion and PROSPERO registration, and has been prepared in accordance with the PRISMA 2020 for Abstracts checklist |
| **INTRODUCTION** | | |  |
| Rationale | 3 | Describe the rationale for the review in the context of existing knowledge. | Section 1 (Introduction), pages 1-2 |
| Objectives | 4 | Provide an explicit statement of the objective(s) or question(s) the review addresses. | Section 1 (Introduction), page 2, final paragraph; Section 2.1 (Aim of the Review), page 2 |
| **METHODS** | | |  |
| Eligibility criteria | 5 | Specify the inclusion and exclusion criteria for the review and how studies were grouped for the syntheses. | Section 2.2 (Study Inclusion Criteria and PICO Framework), page 2; Section 2.6 (Data Synthesis), page 4 |
| Information sources | 6 | Specify all databases, registers, websites, organisations, reference lists and other sources searched or consulted to identify studies. Specify the date when each source was last searched or consulted. | Section 2.3 (Search Strategy), pages 2-3. PubMed (MEDLINE), the Cochrane Library and ClinicalTrials.gov were searched, with a final search date of 30 January 2026 |
| Search strategy | 7 | Present the full search strategies for all databases, registers and websites, including any filters and limits used. | Section 2.3 (Search Strategy), pages 2-3; Fig. 1 (PRISMA flow diagram), page 3. The full search strategy is available on reasonable request |
| Selection process | 8 | Specify the methods used to decide whether a study met the inclusion criteria of the review, including how many reviewers screened each record and each report retrieved, whether they worked independently, and if applicable, details of automation tools used in the process. | Section 2.3 (Search Strategy), pages 2-3, and Section 2.4, page 3. Two reviewers independently screened the literature; discrepancies were resolved between them and, if necessary, by a third reviewer |
| Data collection process | 9 | Specify the methods used to collect data from reports, including how many reviewers collected data from each report, whether they worked independently, any processes for obtaining or confirming data from study investigators, and if applicable, details of automation tools used in the process. | Section 2.4 (Data Extraction), page 3. Data were independently extracted by two reviewers using a standardized form |
| Data items | 10a | List and define all outcomes for which data were sought. Specify whether all results that were compatible with each outcome domain in each study were sought (e.g. for all measures, time points, analyses), and if not, the methods used to decide which results to collect. | Section 2.4 (Data Extraction), page 3, and Section 2.6 (Data Synthesis), page 4. Outcomes sought were a composite of stroke, transient ischemic attack or systemic embolism, major bleeding (ISTH or study-defined) and all-cause mortality. For each outcome the results reported at the longest available follow-up in each study were extracted, together with the time point and the measurement definition used by the study authors |
|  | 10b | List and define all other variables for which data were sought (e.g. participant and intervention characteristics, funding sources). Describe any assumptions made about any missing or unclear information. | Section 2.4 (Data Extraction), page 3. Study design, country, sample size, age, sex, comorbidities, intervention and comparator details and follow-up duration were extracted. Where data were missing or unclear, supplementary materials were searched and the gap was recorded; no assumptions were made and study authors were not contacted |
| Study risk of bias assessment | 11 | Specify the methods used to assess risk of bias in the included studies, including details of the tool(s) used, how many reviewers assessed each study and whether they worked independently, and if applicable, details of automation tools used in the process. | Section 2.5 (Quality Assessment), pages 3-4. The Cochrane Risk of Bias 2 (RoB 2) tool and the Newcastle-Ottawa Scale (NOS) were applied independently by two reviewers |
| Effect measures | 12 | Specify for each outcome the effect measure(s) (e.g. risk ratio, mean difference) used in the synthesis or presentation of results. | Section 2.4, page 3, and Section 2.6, page 4; Table 4, pages 11-13; Fig. 2, page 15. Effect measures are hazard ratios as reported by the included studies, and risk ratios for the pooled analyses |
| Synthesis methods | 13a | Describe the processes used to decide which studies were eligible for each synthesis (e.g. tabulating the study intervention characteristics and comparing against the planned groups for each synthesis (item #5)). | Section 2.6 (Data Synthesis), page 4. Studies were grouped by treatment regimen compared and by CKD stage; the four randomized trials of factor Xa inhibitors versus VKAs in dialysis-dependent patients were identified as the one clinically homogeneous set eligible for quantitative synthesis. Study characteristics are presented in Tables 1-3, pages 5-9 |
|  | 13b | Describe any methods required to prepare the data for presentation or synthesis, such as handling of missing summary statistics, or data conversions. | Section 2.6 (Data Synthesis), page 4. No conversions were performed; all data were extracted as reported in the original studies. For the three-arm trial by De Vriese et al., the two rivaroxaban arms were combined and compared with the single VKA arm |
|  | 13c | Describe any methods used to tabulate or visually display results of individual studies and syntheses. | Tables 1-4, pages 5-13; Fig. 1 (PRISMA flow diagram), page 3; Fig. 2 (forest plots), page 15. The risk-of-bias assessment is presented in the Supplementary Material |
|  | 13d | Describe any methods used to synthesize results and provide a rationale for the choice(s). If meta-analysis was performed, describe the model(s), method(s) to identify the presence and extent of statistical heterogeneity, and software package(s) used. | Section 2.6 (Data Synthesis), page 4. Evidence was synthesized narratively for most comparisons owing to clinical and methodological heterogeneity. For the four randomized trials of factor Xa inhibitors versus VKAs in dialysis-dependent patients, risk ratios with 95% CIs were pooled using an inverse-variance random-effects model (DerSimonian-Laird estimator), with a 0.5 continuity correction applied to studies containing a zero-event cell. Heterogeneity was quantified using the I2 statistic and Cochran's Q test. Analyses were performed in Python 3.12.3 using NumPy 2.4.4 and SciPy 1.17.1 |
|  | 13e | Describe any methods used to explore possible causes of heterogeneity among study results (e.g. subgroup analysis, meta-regression). | Section 2.6, page 4, and Section 3.5.3, pages 14 and 16. Statistical heterogeneity in the quantitative synthesis was quantified using the I2 statistic and Cochran's Q test. Formal subgroup analysis and meta-regression were not undertaken; potential sources of variation between studies, such as differences in anticoagulant regimens, study populations, follow-up duration and outcome or bleeding definitions, were explored narratively (Sections 3.5.2 and 4, pages 14 and 16-18, and Section 5, page 18) |
|  | 13f | Describe any sensitivity analyses conducted to assess robustness of the synthesized results. | Section 2.6, page 4, and Section 3.5.3, pages 14 and 16. Sensitivity analyses comprised the Mantel-Haenszel method, the Hartung-Knapp small-sample adjustment, and a post-hoc analysis restricted to the two trials judged to be at low risk of bias |
| Reporting bias assessment | 14 | Describe any methods used to assess risk of bias due to missing results in a synthesis (arising from reporting biases). | Section 2.6, page 4, and Section 3.6 (Risk of Bias Due to Missing Results), page 16. Published reports were cross-referenced with trial registries and study protocols to identify selective non-reporting of critical endpoints |
| Certainty assessment | 15 | Describe any methods used to assess certainty (or confidence) in the body of evidence for an outcome. | Section 2.5 (Quality Assessment), pages 3-4, and Section 3.2 (Risk of Bias Assessment), page 4 |
| **RESULTS** | | |  |
| Study selection | 16a | Describe the results of the search and selection process, from the number of records identified in the search to the number of studies included in the review, ideally using a flow diagram. | Fig. 1 (PRISMA flow diagram), page 3; Section 2.3, pages 2-3; Section 3.1 (Study Characteristics), page 4 |
|  | 16b | Cite studies that might appear to meet the inclusion criteria, but which were excluded, and explain why they were excluded. | Fig. 1 (PRISMA flow diagram), page 3, which reports the number of reports excluded at full-text assessment together with the reasons for exclusion (study design, ineligible intervention, ineligible population) |
| Study characteristics | 17 | Cite each included study and present its characteristics. | Section 3.1 (Study Characteristics), page 4; Tables 1-3, pages 5-9 |
| Risk of bias in studies | 18 | Present assessments of risk of bias for each included study. | Section 3.2 (Risk of Bias Assessment), page 4; Fig. 2 legend, page 15. The full domain-level assessment is provided in the Supplementary Material (page 19) |
| Results of individual studies | 19 | For all outcomes, present, for each study: (a) summary statistics for each group (where appropriate) and (b) an effect estimate and its precision (e.g. confidence/credible interval), ideally using structured tables or plots. | Table 4, pages 11-13; Fig. 2, page 15, which presents event counts and study-specific risk ratios with 95% CIs for each trial in the quantitative synthesis |
| Results of syntheses | 20a | For each synthesis, briefly summarise the characteristics and risk of bias among contributing studies. | Section 3.5.3, pages 14 and 16; Tables 1-3, pages 5-9; Section 3.2, page 4. Of the four trials contributing to the synthesis, two were judged to be at low risk of bias and two to raise some concerns |
|  | 20b | Present results of all statistical syntheses conducted. If meta-analysis was done, present for each the summary estimate and its precision (e.g. confidence/credible interval) and measures of statistical heterogeneity. If comparing groups, describe the direction of the effect. | Section 3.5.3 (Quantitative Synthesis), pages 14 and 16; Fig. 2, page 15. Pooled risk ratios with 95% CIs and I2 are reported for ISTH major bleeding (RR 0.64, 95% CI 0.42-0.99; I2 = 0%), the composite of stroke, transient ischemic attack or systemic embolism (RR 0.46, 95% CI 0.20-1.02; I2 = 0%) and all-cause mortality (RR 0.88, 95% CI 0.58-1.35; I2 = 46%), each favouring or neutral with respect to factor Xa inhibitors as described in the text |
|  | 20c | Present results of all investigations of possible causes of heterogeneity among study results. | Section 3.5.3, pages 14 and 16 (I2 and Cochran's Q for each synthesis). No formal subgroup analysis or meta-regression was performed owing to the small number of trials; differences between studies were explored narratively in Sections 3.5.2 and 4, pages 14 and 16-18, and in Section 5, page 18 |
|  | 20d | Present results of all sensitivity analyses conducted to assess the robustness of the synthesized results. | Section 3.5.3, pages 14 and 16. The Mantel-Haenszel and Hartung-Knapp analyses attenuated the major bleeding estimate to RR 0.67 (95% CI 0.44-1.03) and RR 0.64 (95% CI 0.34-1.21) respectively. In the post-hoc analysis restricted to the two trials at low risk of bias, the direction and magnitude of all three estimates were preserved |
| Reporting biases | 21 | Present assessments of risk of bias due to missing results (arising from reporting biases) for each synthesis assessed. | Section 3.6 (Risk of Bias Due to Missing Results), page 16. No significant discrepancies were identified between intended methods and reported results; funnel plots were deemed inappropriate owing to the heterogeneity of the included studies |
| Certainty of evidence | 22 | Present assessments of certainty (or confidence) in the body of evidence for each outcome assessed. | Section 3.2, page 4, and Section 5 (Limitations), page 18. Most included studies were judged to be at low risk of bias and overall confidence in the findings is moderate. A formal GRADE assessment was not undertaken; the certainty of the pooled estimates is discussed narratively, with particular reference to sparse event numbers and wide confidence intervals |
| **DISCUSSION** | | |  |
| Discussion | 23a | Provide a general interpretation of the results in the context of other evidence. | Section 4 (Discussion), pages 16-18 |
|  | 23b | Discuss any limitations of the evidence included in the review. | Section 5 (Limitations), page 18 |
|  | 23c | Discuss any limitations of the review processes used. | Section 5 (Limitations), page 18. Embase was not searched owing to restricted institutional access; data extraction relied on published results and study authors were not contacted for missing information; the review was restricted to English-language publications; and most comparisons were synthesized narratively, which may be more prone to interpretation bias than quantitative methods |
|  | 23d | Discuss implications of the results for practice, policy, and future research. | Section 6 (Conclusion), page 18 |
| **OTHER INFORMATION** | | |  |
| Registration and protocol | 24a | Provide registration information for the review, including register name and registration number, or state that the review was not registered. | Abstract, page 1. PROSPERO Registration: https://www.crd.york.ac.uk/PROSPERO/view/CRD420261286449 |
|  | 24b | Indicate where the review protocol can be accessed, or state that a protocol was not prepared. | Abstract, page 1. The protocol is registered and can be accessed on the PROSPERO database |
|  | 24c | Describe and explain any amendments to information provided at registration or in the protocol. | The protocol registered with PROSPERO (CRD420261286449) specified a narrative synthesis of the included evidence. During peer review, the addition of a quantitative synthesis was suggested. A random-effects meta-analysis was therefore performed post hoc for the single clinically homogeneous comparison identified, namely the four randomized trials of factor Xa inhibitors versus VKAs in dialysis-dependent patients (Section 2.6, page 4, and Section 3.5.3, pages 14 and 16). This analysis, together with the Mantel-Haenszel and Hartung-Knapp sensitivity analyses and the analysis restricted to trials at low risk of bias, was not prespecified at registration and should be regarded as exploratory rather than confirmatory. All other comparisons were synthesized narratively, as planned. No further amendments were made to the registered protocol. |
| Support | 25 | Describe sources of financial or non-financial support for the review, and the role of the funders or sponsors in the review. | Funding, page 18. This research received no external funding |
| Competing interests | 26 | Declare any competing interests of review authors. | Conflicts of Interest, page 18. The authors declare no conflicts of interest |
| Availability of data, code and other materials | 27 | Report which of the following are publicly available and where they can be found: template data collection forms; data extracted from included studies; data used for all analyses; analytic code; any other materials used in the review. | Availability of Data and Materials, page 18; Supplementary Material, page 19. The protocol can be accessed on the PROSPERO database. Detailed methods for the literature search and the risk-of-bias assessment are provided in the Supplementary Material |

*From:*  Page MJ, McKenzie JE, Bossuyt PM, Boutron I, Hoffmann TC, Mulrow CD, et al. The PRISMA 2020 statement: an updated guideline for reporting systematic reviews. BMJ 2021;372:n71. doi: 10.1136/bmj.n71

For more information, visit: <http://www.prisma-statement.org/>
