# Supplementary material for: Oral Anticoagulants in Patients With Atrial Fibrillation and Advanced or End-Stage Renal Disease: A Systematic Review and Meta-Analysis
Source: Rev Cardiovasc Med. 2026 Sep 16;27(9):52237. doi: 10.31083/RCM52237 (PMC13653193; doi:10.31083/RCM52237)
Supplement: Supplementary file 1 [file 2153-8174-27-9-52237-s1.zip › Risk of Bias (RoB-2).pdf]

Trial	Randomization process	Deviations from intended interventions	Missing outcome data	Measurement of the outcome	Selection of the reported result	Overall bias
Pokorney 2022	Low	Low	Some concerns	Low	Low	Some concerns
Reinecke 2023	Low	Low	Low	Low	Low	Low
De Vriese 2021	Low	Low	Low	Low	Low	Low
Stanifer 2020	Low	Low	Low	Low	Some concerns	Some concerns
Harel 2025	Low	Low	Some concerns	Low	Low	Some concerns
