# Supplementary material for: Oral Anticoagulants in Patients With Atrial Fibrillation and Advanced or End-Stage Renal Disease: A Systematic Review and Meta-Analysis
Source: Rev Cardiovasc Med. 2026 Sep 16;27(9):52237. doi: 10.31083/RCM52237 (PMC13653193; doi:10.31083/RCM52237)
Supplement: Supplementary file 1 [file 2153-8174-27-9-52237-s1.zip › Risk of bias assessment NOS.pdf]

Trial	Selection	Selection	Selection	Selection	Comparability	Comparability	Outcome	Outcome	Outcome	NOS Score
	Representativeness of exposed cohort	Selection of non-exposed cohort	Ascertainment of Exposure	Outcome not present at start of study	Control of Confounders	Control for additional factors	Assessment of outcome	Follow up long enough	Adequacy of follow up of cohorts	Stars
Ballegaard 2024	*	*	*	*	*	*	*	*	-	8
Hsu 2023	*	*	-	*	*	*	*	*	-	7
Roh 2024	*	*	-	*	*	*	*	*	-	7
Phan 2019	*	*	-	*	*	*	*	*	-	7
Moore 2024	*	*	-	*	*	-	*	*	-	6
Siontis 2018	*	*	*	*	*	*	*	*	-	8
Soriano 2018	*	*	*	*	*	*	*	-	-	7
Weir 2020	*	*	*	*	*	*	*	*	*	9
Yodogawa 2015	*	*	*	-	-	-	*	*	-	5
Mavrakanas 202[illegible]	*	*	*	*	*	*	*	*	-	8
Yoon 2017	*	*	*	*	*	*	*	*	-	8
Genovesi 2016	*	*	*	*	*	*	*	*	-	8
Chan 2015	*	*	*	-	*	*	*	*	-	7
Wakasugi 2014	*	*	*	*	*	*	*	*	-	8
Shah 2014	*	*	*	*	*	*	*	*	-	8
